# Evolution of fruit development genes in flowering plants

**DOI:** 10.3389/fpls.2014.00300

**Published:** 2014-06-26

**Authors:** Natalia Pabón-Mora, Gane Ka-Shu Wong, Barbara A. Ambrose

**Affiliations:** ^1^Instituto de Biología, Universidad de AntioquiaMedellín, Colombia; ^2^The New York Botanical GardenBronx, NY, USA; ^3^Department of Biological Sciences, University of AlbertaEdmonton, AB, Canada; ^4^Department of Medicine, University of AlbertaEdmonton, AB, Canada; ^5^BGI-Shenzhen, Beishan Industrial ZoneShenzhen, China

**Keywords:** AGAMOUS, INDEHISCENT, FRUITFULL, Fruit development, REPLUMLESS, SPATULA, SHATTERPROOF

## Abstract

The genetic mechanisms regulating dry fruit development and opercular dehiscence have been identified in *Arabidopsis thaliana*. In the bicarpellate silique, valve elongation and differentiation is controlled by *FRUITFULL* (*FUL*) that antagonizes *SHATTERPROOF1-2* (*SHP1*/*SHP2*) and *INDEHISCENT* (*IND*) at the dehiscence zone where they control normal lignification. *SHP1/2* are also repressed by *REPLUMLESS* (*RPL*), responsible for replum formation. Similarly, *FUL* indirectly controls two other factors *ALCATRAZ* (*ALC*) and *SPATULA* (*SPT*) that function in the proper formation of the separation layer. FUL and SHP1/2 belong to the MADS-box family, IND and ALC belong to the bHLH family and RPL belongs to the homeodomain family, all of which are large transcription factor families. These families have undergone numerous duplications and losses in plants, likely accompanied by functional changes. Functional analyses of homologous genes suggest that this network is fairly conserved in Brassicaceae and less conserved in other core eudicots. Only the MADS box genes have been functionally characterized in basal eudicots and suggest partial conservation of the functions recorded for Brassicaceae. Here we do a comprehensive search of *SHP, IND, ALC, SPT*, and *RPL* homologs across core-eudicots, basal eudicots, monocots and basal angiosperms. Based on gene-tree analyses we hypothesize what parts of the network for fruit development in Brassicaceae, in particular regarding direct and indirect targets of *FUL*, might be conserved across angiosperms.

## Introduction

Fruits are novel structures resulting from transformations in the late ontogeny of the carpels that evolved in the flowering plants (Doyle, 2013). Fruits are generally formed from the ovary wall but accessory fruits (e.g., apple and strawberry) may contain other parts of the flower including the receptacle, bracts, sepals, and/or petals (Esau, 1967; Weberling, 1989). For purposes of comparison we will discuss fruits that develop from the carpel wall only. Fruit development generally begins after fertilization when the carpel wall (pericarp) transitions from an ovule containing, often photosynthetic vessel, to a seed containing dispersal unit. The fruit wall will differentiate into endocarp (1-few layers closest to developing seeds, often inner to the vascular bundle), mesocarp (multiple middle layers, including the vascular bundles and outer tissues), and exocarp (for the most part restricted to the outermost layer, and only occasionally including hypodermal tissues) (Richard, 1819; Sachs, 1874; Bordzilowski, 1888; Farmer, 1889; Roth, 1977; Pabón-Mora and Litt, 2011). Fruits are classified by their number of carpels, whether multiple carpels are free or fused, texture (dry or fleshy), how the pericarp layers differentiate and whether and how the fruits open to disperse the seeds contained inside (Roth, 1977).

**Figure 1:**
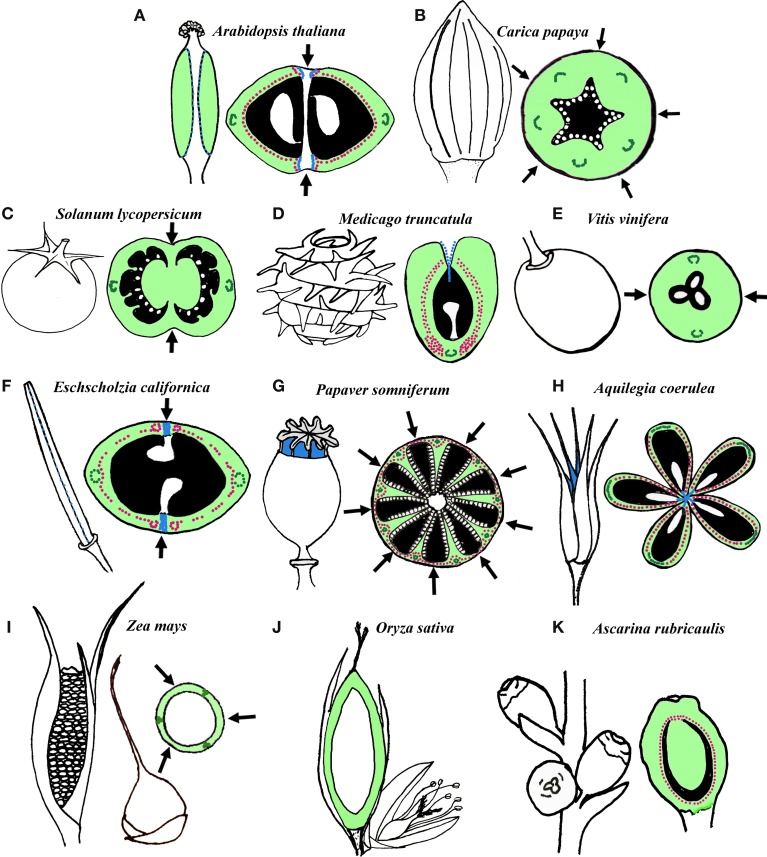
**Schematic representation and transverse/longitudinal sections of several fruits**. **(A–E)** Examples of fruits in core eudicots. **(A)** Operculate capsule of *Arabidopsis thaliana* (Brassicaceae) derived from a bicarpellate and bilocular syncarpic gynoecium. **(B)** Berry of *Carica Papaya* (Caricaceae) derived from a pentacarpellate and unilocular syncarpic gynoecium. **(C)** Berry of *Solanum lycopersicum* (Solanaceae) derived from a bicarpellate and bilocular gynoecium. **(D)** Dehiscent pod of *Medicago truncatula* (Fabaceae) derived from a recurved single carpel. **(E)** Berry of *Vitis vinifera* (Vitaceae) derived from a bicarpellate and unilocular gynoecium. **(F–H)** Examples of fruits in basal eudicots. **(F)** Longitudinally dehiscent capsule of *Eschscholzia californica* (Papaveraceae) derived from a bicarpellate and unilocular syncarpic gynoecium. **(G)** Poricidal capsule of *Papaver somniferum* (Papaveraceae) derived from an 8- to 10-carpellate syncarpic gynoecium with numerous incomplete locules. **(H)** Longitudinally dehiscent follicles of *Aquilegia coerulea* (Ranunculaceae) derived from a pentacarpellate apocarpic gynoecium. **(I–J)** Caryopsis of Poaceae **(I)** *Zea mays* and **(J)** *Oryza sativa*. In both species the fruit is derived from 3 carpels. **(K)** Drupe of *Ascarina rubricaulis* (Chloranthaceae) derived from a unicarpellate gynoecium. (Black, locules; light green, carpel wall; dark green, main carpel vascular bundles; pink, Lignified tissue; blue, dehiscence zones; white, seeds; arrows, fusion between carpels).

There is a vast amount of fruit morphological diversity and fruit terminology that corresponds to this diversity (reviewed in Esau, 1967; Weberling, 1989; Figure 1). For example, fruits made of a single carpel include follicles or pods (e.g., *Medicago truncatula;* Figure 1D) and sometimes drupes (e.g., *Ascarina rubricaulis*; Figure 1K). Follicles and pods both have thick walled exocarp and thin walled parenchyma cells in the mesocarp. However, follicles also have thin walled parenchyma cells in the endocarp while many pods have a heavily sclerified endocarp with 2 distinct layers with microfibrils oriented in different directions (Roth, 1977). When follicles mature the parenchyma and schlerenchyma cell layers dry at different rates causing the fruit to open at the carpel margins (adaxial suture) while pods open at the carpel margin and the median bundle of the carpel due to additional tensions in the endocarp (Roth, 1977; Fourquin et al., 2013). Fruits that are multicarpellate but not fused can include follicles that are free on a receptacle (e.g., *Aquilegia coerulea;* Figure 1H). Fruits that are multi-carpellate and fused include berries (e.g., *Solanum lycopersicum, Carica papaya*, and *Vitis vinifera*; Figures 1B,C,E), capsules (e.g., *Arabidopsis thaliana, Eschscholzia californica, Papaver somniferum*; Figures 1A,F,G), caryopses (grains of *Oryza sativa* and *Zea mays*; Figures 1I,J), and drupes (e.g., peach). These multicarpellate fruits differ by the differentiation of the pericarp and their dehiscence mechanisms. Berries and drupes tend to be indehiscent and the pericarp of berries is often fleshy and composed mainly of parenchyma tissue (Richard, 1819; Roth, 1977). The endocarp and mesocarp of drupes is also fleshy, however, the endocarp is composed of highly sclerified tissue termed the stone (Richard, 1819; Sachs, 1874). Caryopses are also indehiscent and have a thin wall of pericarp fused to a single seed (Roth, 1977). Capsules can have few to many cells in the pericarp and the different layers of the pericarp can be composed of parenchyma tissue in most layers and sclerenchyma tissue in the mesocarp and/or endocarp. Capsules can dehisce at various locations including at the carpel margins (septicidal), at the median bundles (loculicidal) or through small openings (poricidal) (Roth, 1977). The extreme fruit morphologies found across angiosperms, even in closely related taxa suggest that fruits are an adaptive trait, thus, homoplasious seed dispersal forms and transformations from berries to capsules or drupes and vice versa are common in many plant families (Pabón-Mora and Litt, 2011).

The molecular basis that underlies fruit diversity is not well-understood. However, the fruit molecular genetic network in *Arabidopsis thaliana* (Arabidopsis), necessary to specify the different components of the fruit including the sclerified (lignified) tissues necessary for the controlled opening (dehiscence) of the fruit are well-characterized (Reviewed in Ferrándiz, 2002; Roeder and Yanofsky, 2006; Seymour et al., 2013). Arabidopsis fruits develop from two fused carpels and are specialized capsules called siliques, which open along a well-defined dehiscence zone (Hall et al., 2002: Avino et al., 2012). The siliques are composed of two valves separated by a unique tissue termed the replum present only in the Brassicaceae. The valves develop from the carpel wall and are composed of an endocarp, mesocarp and exocarp. The replum and valves are joined together by the valve margin. The valve margin is composed of a separation layer closest to the replum and liginified tissue closer to the valve. The endocarp of the valves becomes lignified late in development and plays a role, along with the lignified layer and separation layer of the valve margin, in fruit dehiscence (Ferrándiz, 2002).

Developmental genetic studies in *Arabidopsis thaliana* have uncovered the genetic network that patterns the Arabidopsis fruit. FRUITFULL (FUL) is necessary for proper valve development and represses SHATTERPROOF 1/2 (SHP 1/2) (Gu et al., 1998; Ferrándiz et al., 2000a). SHP1/2 are necessary for valve margin development (Liljegren et al., 2000). REPLUMLESS (RPL) is necessary for replum development and represses SHP1/2 (Roeder et al., 2003). The repression of SHP1/2 by FUL and RPL keeps valve margin identity to a small strip of cells. SHP1/2 activate INDEHISCENT (IND) and ALCATRAZ (ALC), which are both necessary for the differentiation of the dehiscence zone between the valves and replum (Girin et al., 2011; Groszmann et al., 2011). IND is important for lignification of cells in the dehiscence zone while IND and ALC are necessary for proper differentiation of the separation layer (Rajani and Sundaresan, 2001; Liljegren et al., 2004: Arnaud et al., 2010). SPATULA (SPT) also plays a minor role, redundantly with its paralog ALC in the specification of the fruit dehiscence zone (Alvarez and Smyth, 1999; Heisler et al., 2001; Girin et al., 2010, 2011; Groszmann et al., 2011).

FUL, SHP1/2, RPL, IND, SPT, and ALC all belong to large transcription factor families. FUL and SHP1/2 belong to the MADS-box family (Gu et al., 1998; Liljegren et al., 2000), IND, SPT, and ALC belong to the bHLH family and RPL belongs to the homeodomain family (Heisler et al., 2001; Rajani and Sundaresan, 2001; Roeder et al., 2003; Liljegren et al., 2004). Some of these transcription factors are known to be the result of Brassicaceae specific duplications, others seem to be the result of duplications coinciding with the origin of the core eudicots (Jiao et al., 2011). For instance *SHP1* and *SHP2* are *AGAMOUS* paralogs and Brassicaceae-specific duplicates belonging to the C-class gene lineage (Kramer et al., 2004). FUL is a member of the *AP1/FUL* gene lineage unique to angiosperms (Purugganan et al., 1995). FUL belongs to the euFULI clade, that together with euFULII and euAP1 are core-eudicot specific paralogous clades. Nevertheless, pre-duplication proteins are similar to euFUL proteins, hence they have been named FUL-like proteins and are present in all other angiosperms (Litt and Irish, 2003). Likewise, *ALC* and *SPT* and *IND* are the result of several duplications in different groups of the bHLH family of transcription factors, but the exact duplication points have not yet been identified (Reymond et al., 2012; Kay et al., 2013). Hence, it is unclear whether this gene regulatory network can be extrapolated to fruits outside of the Brassicaceae. Functional evidence from *Anthirrhinum* (Plantaginaceae) (Müller et al., 2001), *Solanum* (Solanaceae) (Bemer et al., 2012; Fujisawa et al., 2014), and *Vaccinium* (Ericaceae) (Jaakola et al., 2010) in the core eudicots, as well as *Papaver* and *Eschscholzia* (Papaveraceae, basal eudicots) (Pabón-Mora et al., 2012, 2013b) suggest that at least *FUL* orthologs have a conserved role in regulating proper fruit development even in fruits with diverse morphologies. *euFUL* and *FUL-like* genes control proper pericarp cell division and elongation, endocarp identity, and promote proper distribution of bundles and lignified patches after fertilization. However, functional orthologs of *SHP, IND, ALC, SPT*, or *RPL* have been less studied and it is unclear whether they are conserved in core and non-core eudicots. The limited functional data gathered suggests that at least in other core eudicots *SHP* orthologs play roles in capsule dehiscence (Fourquin and Ferrandiz, 2012) and berry ripening (Vrebalov et al., 2009). Likewise, *SPT* orthologs have been identified as potential key players during pit formation in drupes, likely regulating proper endocarp margin development (Tani et al., 2011). *RPL* orthologs have not been characterized in core eudicots, but an *RPL* homolog in rice is a domestication gene involved in the non-shattering phenotype, suggesting that the same genes are important to shape seed dispersal structures in widely divergent species (Arnaud et al., 2011; Meyer and Purugganan, 2013). At this point, more expression and functional data are urgently needed to test whether the network is functionally conserved across angiosperms, nevertheless, all these transcription factors are candidate regulators of proper fruit wall growth, endocarp and dehiscence zone identity, and carpel margin identity and fusion (Kourmpetli and Drea, 2014). In the meantime, another approach to study the putative conservation of the network is to identify how these specific gene families have evolved in flowering plants as duplication and diversification of transcription factors are thought to be important for morphological evolution. Although, based on gene analyses no functions can be explicitly identified, the presence and copy number of these genes will provide testable hypothesis for future studies in different angiosperm groups. Thus, to better understand the diversity of fruits and the changes in the fruit core genetic regulatory network we analyzed the evolution of these transcription factor families from across the angiosperms. We utilized data in publicly available databases and performed phylogenetic analyses. We found different patterns of duplication across the different transcription factor families and discuss the results in the context of the evolution of a developmental network across flowering plants.

## Materials and methods

### Cloning and characterization of genes involved in the fruit developmental network

For each of the gene families, searches were performed by using the Arabidopsis sequences as a query to identify a first batch of homologs using Blast tools (Altschul et al., 1990) through Phytozome (http://www.phytozome.net/; Joint Genome Institute, 2010) from all plant genomes available from Brassicaceae and other core eudicots, *Aquilegia coerulea* (basal eudicot) and monocots. To better understand the evolution of the fruit developmental network we have extended our search to other core eudicots, basal eudicots, monocots, basal angiosperms, and gymnosperms using the 1 kp transcriptome database (http://218.188.108.77/Blast4OneKP/home.php). This is a database that comprises more than1000 transcriptomes of green plants and therefore represents a large dataset for blasting orthologous genes of the core fruit gene network outside of Brassicaceae. It is important to note that the oneKP public blast portal does not have the complete transcriptomes publicly available yet for many species and that often the transcriptomes available are those from leaf tissue, reducing the possibilities to blast fruit specific genes in some taxa. In addition we used two additional databases: The Ancestral Angiosperm Genome Project (AAGP) http://ancangio.uga.edu to search specific sequences in *Aristolochia* (Aristolochiaceae, basal angiosperms) and *Liriodendron* (Magnoliaceae, basal angiosperms) and Phytometasyn (http://www.phytometasyn.ca) to search specific sequences from basal eudicots. The sampling was specifically directed to seed plants, therefore outgroup sequences included homologs of ferns and mosses of the targeted gene family (when possible) in addition to closely related gene groups (Supplementary Tables 1–5). Outgroup sequences used for the *APETALA1*/*FRUITFULL* genes include *AGAMOUS Like-6* genes from several angiosperms (Litt and Irish, 2003; Zahn et al., 2005; Viaene et al., 2010). For *AGAMOUS/SEEDSTICK* genes the outgroup includes *AGAMOUS Like-12* sequences from several angiosperms (Becker and Theissen, 2003; Carlsbecker et al., 2013). For *HECATE3*/*INDEHISCENT* genes outgroup sequences include the closely related AtbHLH52 and AtbHLH53 from *Arabidopsis* as well has *HECATE1* and *HECATE2* from other angiosperms (Heim et al., 2003; Toledo-Ortiz et al., 2003). For *SPATULA/ALCATRAZ* outgroup sequences include *HEC3*/*IND* from *Arabidopsis* and other angiosperms (Heim et al., 2003; Toledo-Ortiz et al., 2003; Reymond et al., 2012), and finally for *REPLUMLESS/POUND-FOOLISH* genes the outgroup sequences include *AtSAW1, AtSAW2*, and *AtBEL1*, as well as *SAW1* and *SAW2* angiosperm homologs (Kumar et al., 2007; Mukherjee et al., 2009). Vouchers of all sequences and accession numbers are supplied in Supplementary Tables 1–5.

### Phylogenetic analyses

**Figure 2:**
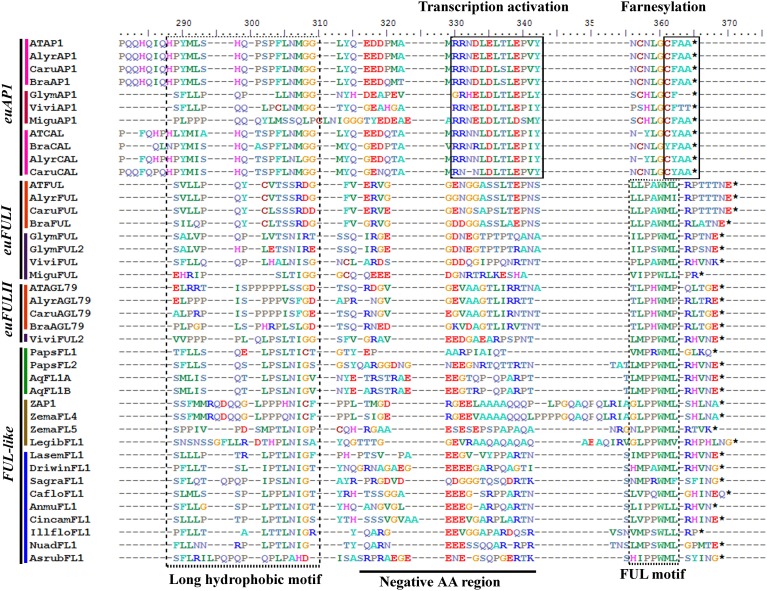
**Alignment of the end of the K and the complete C-terminal domain of APETALA1/FRUITFULL proteins (labeled with the clade names they belong to)**. Colors to the left of the sequences indicate the taxon they belong to as per color key in Figure 3. The box to the left shows a conserved long hydrophobic motif, previously identified, but with unknown function, followed by a region variable but consistently with negatively charged amino acids [i.e., rich in glutamic acid (E) particularly in euFULI, euFULII, and FUL-like proteins, and in arginine (R), particularly in euAP1 proteins]. The transcription activation and the farnesylation motifs (boxed) distinguish the euAP1 proteins. The FUL-motif (boxed) is typically found in FUL-like and euFUL proteins.

Sequences in the transcriptome databases were compiled using Bioedit (http://www.mbio.ncsu.edu/bioedit/bioedit.html), where they were cleaned to keep exclusively the open reading frame. Nucleotide sequences were then aligned using the online version of MAFFT (http://mafft.cbrc.jp/alignment/server/) (Katoh et al., 2002), with a gap open penalty of 3.0, an offset value of 0.8, and all other default settings. The alignment was then refined by hand using Bioedit taking into account the protein domains and amino acid motifs that have been reported as conserved for the five gene lineages (alignments shown in Figures 2, **4**, **6**, **8**, **10**) Maximum Likelihood (ML) phylogenetic analyses using the nucleotide sequences were performed in RaxML-HPC2 BlackBox (Stamatakis et al., 2008) on the CIPRES Science Gateway (Miller et al., 2009). The best performing evolutionary model was obtained by the Akaike information criterion (AIC; Akaike, 1974) using the program jModelTest v.0.1.1 (Posada and Crandall, 1998). Bootstrapping was performed according to the default criteria in RAxML where bootstrapping stopped after 200–600 replicates when the criteria were met. Trees were observed and edited using FigTree v1.4.0. Uninformative characters were determined using Winclada Asado 1.62.

## Results

### *APETALA1*/*FRUITFULL* gene lineage

APETALA1 (AP1) and FRUITFULL (FUL) are members of the *AP1/FUL* gene lineage. Thus, they belong to the large MADS-box gene family present in all land plants (Gustafson-Brown et al., 1994; Purugganan et al., 1995; Gu et al., 1998; Alvarez-Buylla et al., 2000; Becker and Theissen, 2003). Sequences of AP1 and FUL recovered by similarity in the transcriptomes generally span the entire coding sequence, although some are missing 20–30 amino acids (AA) from the start of the 60 AA MADS domain. The alignment includes the conserved MADS (M) and K domains, approximately with 60 AA and 70–80 AA, respectively, an intervening domain (I) between them with 30 and 40 AA and the C-terminal domain of approximately 200 AA. The alignment of the ingroup consists of a total of 180 sequences (i.e., 29 sequences from 25 species of basal angiosperms, 12 sequences from 4 species of monocots, 44 sequences from 22 species of basal eudicots, and 95 sequences from 35 species of core eudicots). Predicted amino acid sequences of the entire dataset reveal a high degree of conservation in the M, I, and K regions until position 222. The C-terminal domain is more variable, but four regions of high similarity can be identified: (1) a region rich in tandem repeats of polar uncharged amino acids (PQN) up until position 285 in the alignment (Moon et al., 1999); (2) a highly conserved, predominantly hydrophobic motif between positions 290 and 310; (3) a negatively charged region rich in glutamic acid (E) that includes the transcription activation motif in euAP1 proteins (Cho et al., 1999) and (4) the end of the protein that includes a farnesylation motif (CF/YAA) for euAP1 proteins (Yalovsky et al., 2000) and the FUL motif (LMPPWML) for euFUL and FUL-like proteins (Litt and Irish, 2003) (Figure 2).

**Figure 3:**
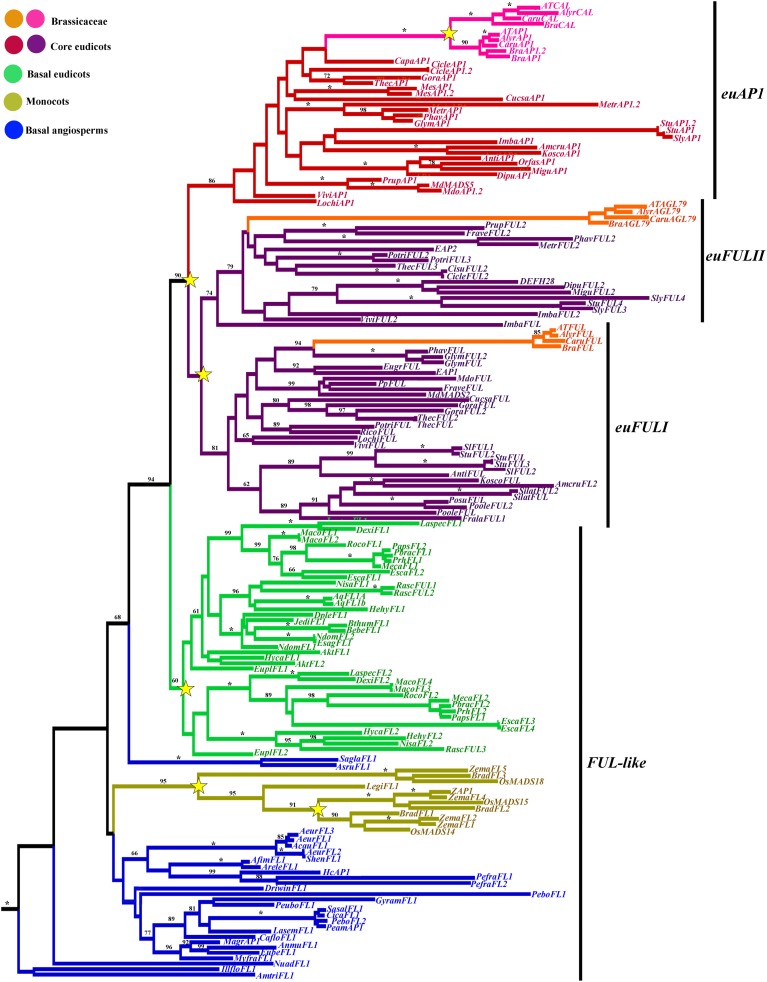
**ML tree of *APETALA1/FRUITFULL* genes in angiosperms showing five duplication events (yellow stars)**. Two duplications in Poaceae, resulting in three distinct monocot *FUL-like* clades; one duplication in basal eudicots resulting in two Ranunculiid *FUL-like* clades; two duplications in the core eudicots resulting in the *euFULI, euFULII*, and *euAP1* clades and one additional duplication specific to Brassicaceae resulting in the *CAL* clade. Branch colors denote taxa as per the color key at the top left; BS values above 50% are placed at nodes; asterisks indicate BS of 100.

A total of 1715 characters were included in the matrix, of which 1117 (65%) were informative. Maximum likelihood analysis recovered five duplication events, two affecting monocots, particularly grasses resulting in *FUL1, FUL2*, and *FUL3* genes (Preston and Kellogg, 2006), another occurring early in the diversification of the Ranunculales in the basal eudicots resulting in the *RanFL1* and *RanFL2* clades (Pabón-Mora et al., 2013b) and two coincident with the diversification of the core-eudicots (Litt and Irish, 2003; Shan et al., 2007) resulting in the *euFULI, euFULII*, and *euAP1* clades (Figure 3). Bootstrap supports (BS) for those clades is above 80 except for the *RanFL1* and *RanFL2* clades, however within each clade, gene copies from the same family are grouped together with strong support (Pabón-Mora et al., 2013b), and the relationships among gene clades are mostly consistent with the phylogenetic relationships of the sampled taxa (Wang et al., 2009). Another duplication occurred concomitantly with the core-eudicot diversification and resulted in the *euAP1* and *euFUL* gene clades (90 BS), followed by another duplication in the *euFUL* clade resulting in the *euFULI* and *euFULII* clades (Figure 3; Litt and Irish, 2003; Shan et al., 2007). The duplication itself has low BS, but the *euFULI* and *euFULII* clades have high support with 81 and 74, respectively. Within Brassicaceae another duplication occurred within the euAP1 clade resulting in the *AP1* and *CAL* Brassicaceae gene clades (100 BS) (Figure 3; Lowman and Purugganan, 1999; Alvarez-Buylla et al., 2006). Major sequence changes are linked with the core-eudicot duplication. Whereas euFUL proteins retain the characteristic FUL-like motif present in FUL-like pre-duplication proteins present in basal angiosperms, monocots and basal eudicots, the euAP1 proteins acquired, due to a frameshift mutation, a transcription activation and a farnesylation motif at the C-terminus (Cho et al., 1999; Yalovsky et al., 2000; Litt and Irish, 2003; Preston and Kellogg, 2006; Shan et al., 2007), that is very conserved in CAL proteins as well Kempin et al. (1995); Alvarez-Buylla et al. (2006).

Taxon-specific *euFUL* duplications have occurred in *Solanum* (Solanaceae), *Theobroma, Gossypium* (Malvaceae), *Eucalyptus* (Myrtaceae), *Glycine* (Fabaceae), *Populus* (Salicaceae) *Portulaca* (Portulacaceae), *Silene* (Caryophyllaceae), and *Malus* (Rosaceae) (Figure 3). On the other hand, *euFUL* homologs are likely to be pseudogenized in *Manihot* (Euphorbiaceae), and *Carica* (Caricaceae), where searches on the available genomic sequences, did not retrieve any *euFUL* orthologs. Taxon-specific *euAP1* duplications have occurred in *Malus* (Rosaceae), *Solanum* (Solanaceae), *Manihot* (Euphorbiaceae), and *Citrus* (Rutaceae). *euAP1* homologs seem to be lacking for *Eucalyptus* (Myrtaceae), as sequences previously reported as *EAP1* and *EAP2* by Kyozuka et al. (1997) are members of the *euFULI* and *euFULII* clades. *euAP1* Homologs were also not found in *Fragaria* (Rosaceae) but have been previously reported (Zou et al., 2012) suggesting that the sequence may be divergent enough that is not found through the phytozome blast search. Similarly, *euAP1* sequences were not found in the transcriptomic sequences available for *Silene* (Caryophyllaceae), but have been found before (SLM4, SLM5; Hardenack et al., 1994). In addition, they are likely missing or silent (not expressed) in *Portulaca* (Portulacaceae) but these data will have to be reevaluated as more transcriptomic data from these species becomes publicly available.

### *AGAMOUS*/*SEEDSTICK* gene lineage

**Figure 4:**
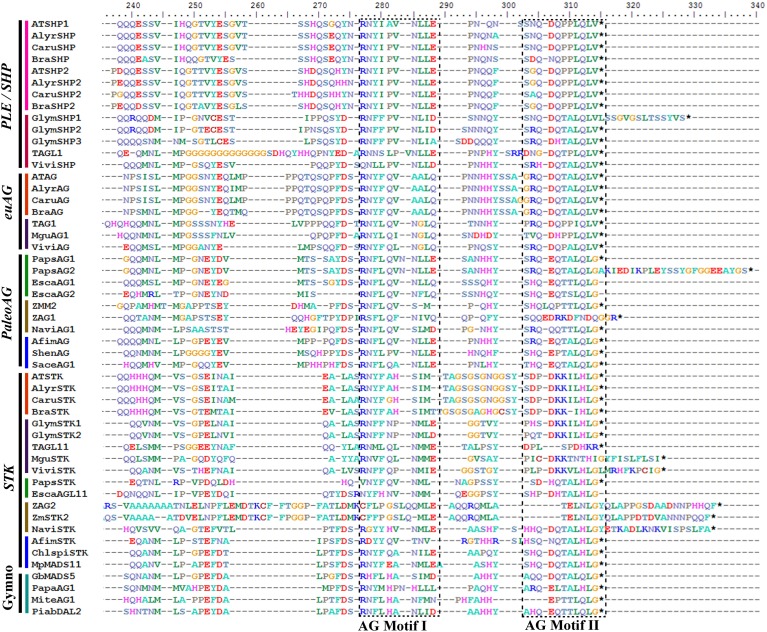
**Alignment of the end of the K and the complete C-terminal domain of AGAMOUS/SEEDTICK proteins (labeled with the clade names they belong to)**. Colors to the left of the sequences indicate the taxon they belong to as per the color key in Figure 5. Previously identified conserved AG Motifs I and II in both protein clades are boxed; note that sequences in between the motifs are very different between the AGAMOUS and the SEEDSTICK orthologous proteins, and there appears to be a GS/GN repeat in this region exclusive to Brassicaceae STK sequences; note also the divergence at the end of the K-domain between the closely related paralogous SHP1 and SHP2 in the Brassicaceae. The alignment also includes the atypical paleoAGAMOUS proteins in *Papaver* (PapsAG1, PapsAG2) due to alternative splicing.

The SEEDSTICK (STK), AGAMOUS (AG), SHATTERPROOF1 (SHP1) and SHP2 proteins belong to the C and D class of the large MADS-box transcription factor family (Yanofsky et al., 1990; Purugganan et al., 1995; Becker and Theissen, 2003; Colombo et al., 2008). Sequences recovered by similarity in the transcriptomes generally span the entire coding sequence, although some are missing 20–30 amino acids (AA) from the start of the 60 AA MADS domain. The alignment includes the conserved MADS and K domains, approximately with 60 AA and 60–80 AA, respectively, an intervening domain between them with 25 and 30 AA and the C-terminal domain expanding ca. 200 AA. The alignment of the ingroup consists of a total of 185 sequences (i.e., 14 sequences from 14 species of gymnosperms, 13 sequences from 11 species of basal angiosperms, 24 sequences from 18 species of monocots, 35 sequences from 18 species of basal eudicots, and 89 sequences from 40 species of core eudicots). Predicted amino acid sequences of the entire dataset reveal a high degree of conservation in the M, I, and K regions until position 228. A few positions conserved that distinguish the STK from the AG/SHP clade such as the typical Q105 always present in the STK proteins (with the exception of ChlspiSTK) (Kramer et al., 2004; Dreni and Kater, 2014). Others that distinguish between the AG and the PLE/SHP clades are the GI or IS in positions 105/106 in euAG proteins vs. the conserved RD in the same positions in PLE/SHP proteins. The C-terminal domain is more variable, but two regions of high similarity can be identified: (1) The AG Motif I and (2) The AG Motif II both with predominantly acidic or hydrophobic amino acids. These two motifs are conserved in both the *AGAMOUS/SHATTERPROOF* and the *SEEDSTICK* gene clades in angiosperms as well as in the pre-duplication gymnosperm homologous genes (Figure 4) (Kramer et al., 2004; Dreni and Kater, 2014). Only Poaceae *AG/SHP* and *STK* homologs present noticeable divergence in those motifs (Figure 4; Dreni and Kater, 2014).

**Figure 5:**
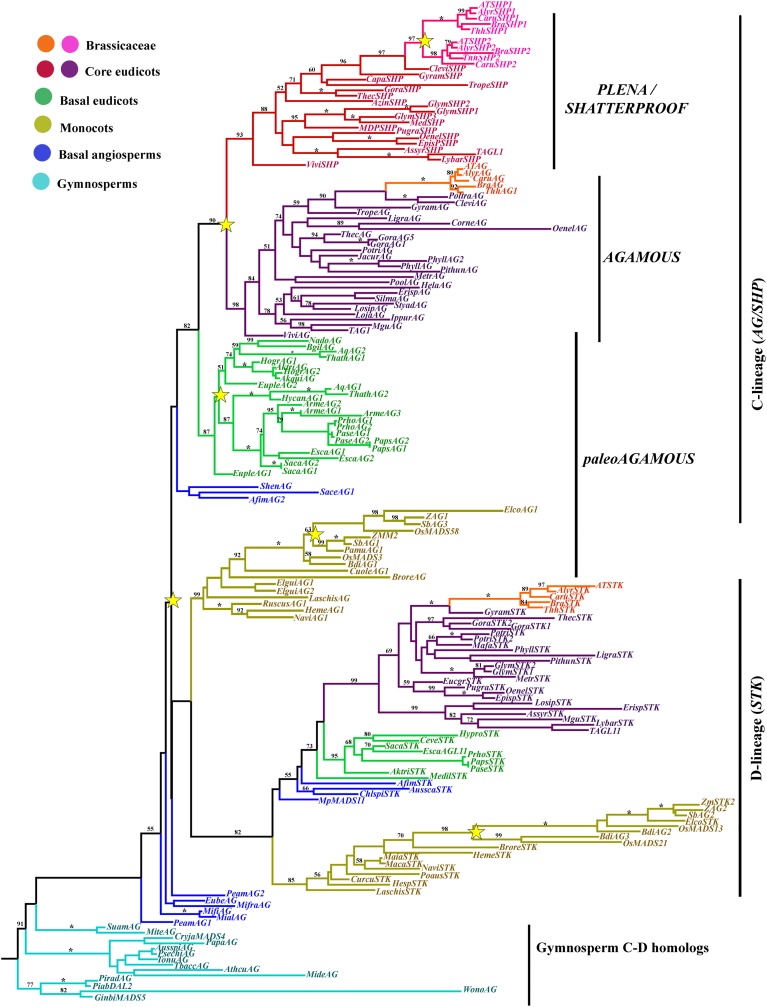
**ML tree of *AGAMOUS/SEEDSTICK* genes in seed plants showing a number of duplication events (yellow stars)**. A duplication coincident with the diversification of the angiosperms, resulting in the D-lineage and the C-lineage clades (also known as *AGL11* and *AG* lineage, respectively). The D-lineage underwent a duplication in Poaceae but for the most part has been kept as single copy in angiosperms (see text for exceptions). The C-lineage duplicated independently in Poaceae, resulting in two paleoAG grass clades, in basal eudicots, resulting in two Ranunculaceae specific clades, and in the core eudicots, resulting in the *euAG* and the *PLE/SHP* gene lineages. An additional duplication occurred with the diversification of the Brassicaceae resulting in the *SHP1* and *SHP2* clades. Branch colors denote taxa as per color key at the top left; BS above 50% are placed at nodes; asterisks indicate BS of 100.

A total of 1720 characters were included in the matrix, of which 915 (53%) were informative. Maximum likelihood analysis recovered five duplication events. The most important one occurred concomitantly with the origin of angiosperms and resulted in the *AG/SHP* and the *STK* gene clades (Figure 5). BS for this duplication is low (<50), and the position of the *AG/SHP* monocot clade is variable (retested in parsimony analyses, data not shown), nevertheless the two main resulting clades have BS of 82 and within each clade, relationships among genes are mostly consistent with the phylogenetic relationships of the sampled taxa (APG, 2009). This contrasts with the single copy C and D class genes found in gymnosperms (Kramer et al., 2004; Carlsbecker et al., 2013). They appear to be paraphyletic with respect to the angiosperm C and D lineages, but the three clades that they form have strong supports (Figure 5). Both angiosperm gene lineages underwent additional duplications in the grasses that for the most part have two *AG/SHP* gene clades and two *STK* gene clades (Dreni et al., 2013). The *STK* genes have remained mostly single copy in all other angiosperms including basal angiosperms and basal and core eudicots, with only two exceptions. In monocots the radiation of the Poaceae seems to be associated with a duplication in the *STK* genes (BS 98), and in the core eudicots, taxon specific duplications seem to have affected independently *Gossypium* (Malvaceae) and *Glycine* (Fabaceae), each with two *STK* paralogs (Figure 5). In addition, our data supports the idea that *STK* genes have been lost or are not expressed in the Eupteleaceae and the Ranunculaceae (basal eudicots), as *STK* homologs were not retrieved from the transcriptomic data available for *Euptelea* or the *Aquilegia* genome. This is consistent with the findings of Liu et al. (2010) and Kramer et al. (2004).

The *AG/SHP* genes have undergone additional duplications during angiosperm diversification. One such duplication seems to have occurred in basal eudicots, before the diversification of the Ranunculaceae, that has two gene clades with strong support (100BS) however, the exact time is unclear as sampling is limited (Figure 5; Yellina et al., 2010). Members of the Papaveraceae, also have two paralogous *AG* genes, however, at least in *Papaver* species and the closely related *Argemone*, the two transcripts seem to be the result of alternative splicing, identical to the case reported in *P. somniferum* by Hands et al. (2011). Two additional duplications occurred in the *AG/SHP* genes, one connected with the diversification of the core eudicots resulting in the *euAG* and the *PLE/SHP* clades (90BS), and the second one in the *PLE/SHP* clade in Brassicaceae resulting in the *SHP1* and *SHP2* gene clades (97BS; Figure 5; Kramer et al., 2004; Zahn et al., 2006).

Taxon-specific *euAG* duplications have occurred in *Gossypium* (Malvaceae) and *Phyllanthus* (Euphorbiaceae). Likewise, *PLE/SHP* specific duplications have affected *Glycine* (Fabaceae) and *Brassica* (Brasicaceae). On the other hand, *euAG* homologs are likely to be pseudogenized or have diverged dramatically in sequence in *Malus* (Rosaceae), *Glycine* (Fabaceae), and *Carica* (Caricaceae), as an exhaustive search in their available genomic sequences did not result in any significant hit. Similarly, *PLE/SHP* homologs have diverged considerably or have been lost in *Populus* (Salicaceae) and *Mimulus* (Phrymaceae). Our analysis did not find any *PLE/SHP* homologs in *Lonicera* (Caprifoliacaeae), *Lobelia* (Campanulaceae), *Stylidium* (Stylidiaceae), *Sylibum, Erigeron* (Asteraceae), *Coriaria* (Coriariaceae), *Heracleum* (Asteraceae), *Polansia* (Capparaceae), *Ipomoea* (Colvolvulaceae), and *Linum* (Linaceae). Some of the same cases were also noticed by Dreni and Kater (2014) (i.e., loss of *euAG* in *Carica*, and loss of *PLE/SHP* in *Populus* and *Mimulus*), suggesting that pseudogenization likely happened in *PLE/SHP* genes of many core eudicots after the duplication event, however these data would have to be confirmed as a larger set of transcripts from these species becomes publicly available. This scenario is very different in Brassicaceae, where additional duplications occurred as a result of a Whole Genome Duplications (WGD) (Barker et al., 2009; Donoghue et al., 2011) but functional paralogs only remained in the *PLE/SHP* clade with two *SHP* homologs. The Brassicaceae specific copies resulting from this duplication in the *euAG* and the *STK* clades have been likely pseudogenized.

### *ALCATRAZ*/*SPATULA* gene lineage

**Figure 6:**
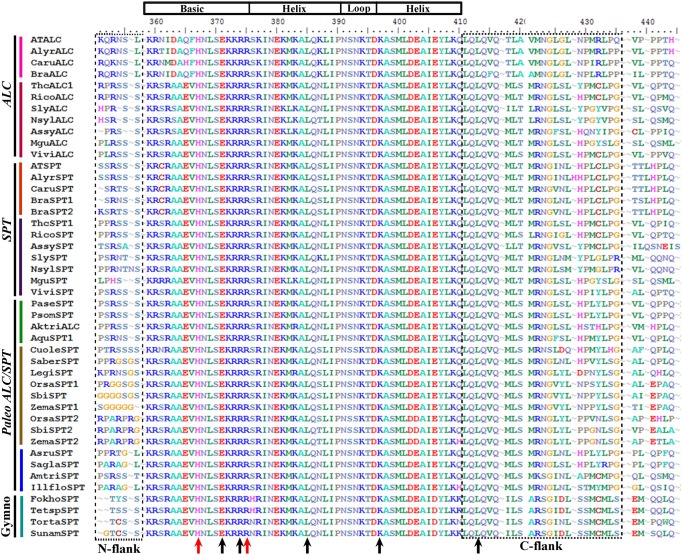
**Alignment of the bHLH domain of SPATULA/ALCATRAZ proteins (labeled with the clade names they belong to)**. Colors to the left of the sequences indicate the taxon they belong to as per color conventions in Figure 7. The bHLH was drawn based on Toledo-Ortiz et al. (2003) and in our alignment corresponds with positions K359-Q410. The alignment shows an N-flank before the start of the bHLH domain rich in Serine (S). Within the bHLH domain, black arrows indicate positions E13, R16, L27, K39, L56, which are conserved in all bHLH plant and animal genes. E13 provides the SPT/ALC proteins with E-box binding (CANNTG) activity. The H9 and R17 positions (red arrows) show aminoacids that provide the SPT/ALC proteins with G-box (CACGTG) binding activity. The alignment also shows the conserved motif LQLQVQ in the C-flank of the bHLH motif followed by a fairly conserved motif MLS/TMRNGLSLH/N/PPL/MGLPG (boxed).

ALCATRAZ (ALC) and SPATULA (SPT) belong to the large bHLH transcription factor family (Toledo-Ortiz et al., 2003; Reymond et al., 2012). Sequences recovered by similarity in the transcriptomes generally span the entire coding sequence. Alignment of the ingroup consists of a total of 139 sequences (i.e., 7 sequences from 7 species of gymnosperms, 5 sequences from 5 species of basal angiosperms, 16 sequences from 13 species of monocots, 14 sequences from 14 species of basal eudicots, and 97 sequences from 53 species of core eudicots). Predicted amino acid sequences of the entire dataset reveal a high degree of conservation in the M, I, and K regions until position 222. The alignment includes a first region extremely variable of 310 AA, where only a few local blocks of conserved amino acids (AA) are observed in closely related species. A second region follows this from 311 to 349 AA with a largely conserved motif DDLDCESEEGG/QE rich in hydrophobic and negative amino acids, in all members of the SPT/ALC proteins in gymnosperms and angiosperms. The exceptions are: The SPT-like2 grass clade with the sequence E/Q H/QLDLVMRHH/Q and the ALC Brassicaceae clade with the sequence VAETS/AQE/DKYA that have more polar uncharged amino acids accompanying the hydrophobic and negatively charged ones (not shown; this region is located immediately before the N-flank shown in Figure 6). Right after this region and before the bHLH domain there is a region from 350 to 357 AA in the alignment, rich in polar uncharged and positively charged amino acids fairly conserved across angiosperms and gymnosperms (R/PS/PRSSS/L) with the exception of the SPT-like1 paralogous grass genes that have instead Glycine (G) repeats in this region, labeled as N-flank in reference to the bHLH domain (Figure 6). Within the bHLH domain that goes from AA 359 to 410, the SPT/ALC proteins as most other AtbHLH proteins have on average 9 positively charged (K, R, and H) amino acids, in the basic motif that spans 17 AA (Figure 6). This is followed by the completely conserved helices interrupted by a loop (HLH), responsible for homodimerization and heterodimerization (Murre et al., 1989; Ferre-D'Amare et al., 1994; Nair and Burley, 2000; Toledo-Ortiz et al., 2003). SPT/ALC share with most other bHLH proteins studied to date, from both animals and plants, the positions H9, E13, R16, L27, K39, L56 (Figure 6). The presence of E13 and R16 makes SPT/ALC proteins E-box binders (CANNTG), as these residues are critical to contact the CA in the E-box and confers the DNA binding activity of SPT/ALC proteins (Fisher and Goding, 1992; Ellenberg et al., 1994; Shimizu et al., 1997; Fuji et al., 2000). Furthermore, the E13 residue is essential for DNA binding. SPT/ALC proteins can be further classified into G-box (CACGTG) binders within the E-box binders category, as they possess the H9, E13, R17 positions (Toledo-Ortiz et al., 2003). This binding, specifically to G-boxes, has been demonstrated *in vitro* for SPT (Reymond et al., 2012). After the end of the second helix there is a conserved motif LQLQVQ completely conserved in all sequences, followed by a fairly conserved motif MLS/TMRNGLSLH/N/PPL/MGLPG, both are included at the C-flank of the bHLH motif. This last motif is once again more variable in the ALC Brassicaceae paralogs and in the gymnosperm SPT/ALC homologs (Figure 6). From the position 438 until the end of the alignment there are no other regions that seem to be conserved across all SPT/ALC homologs, nevertheless there are some small regions that can be confidently aligned, particularly among closely related plant groups. In this region, there is a very noticeable increase in variation and shortening of the coding sequence in the Brassicaceae ALC homologs suggesting a faster sequence mutation rate. This is likely linked with divergent functions in this gene clade compared with other angiosperm and gymnosperm SPT/ALC proteins.

**Figure 7:**
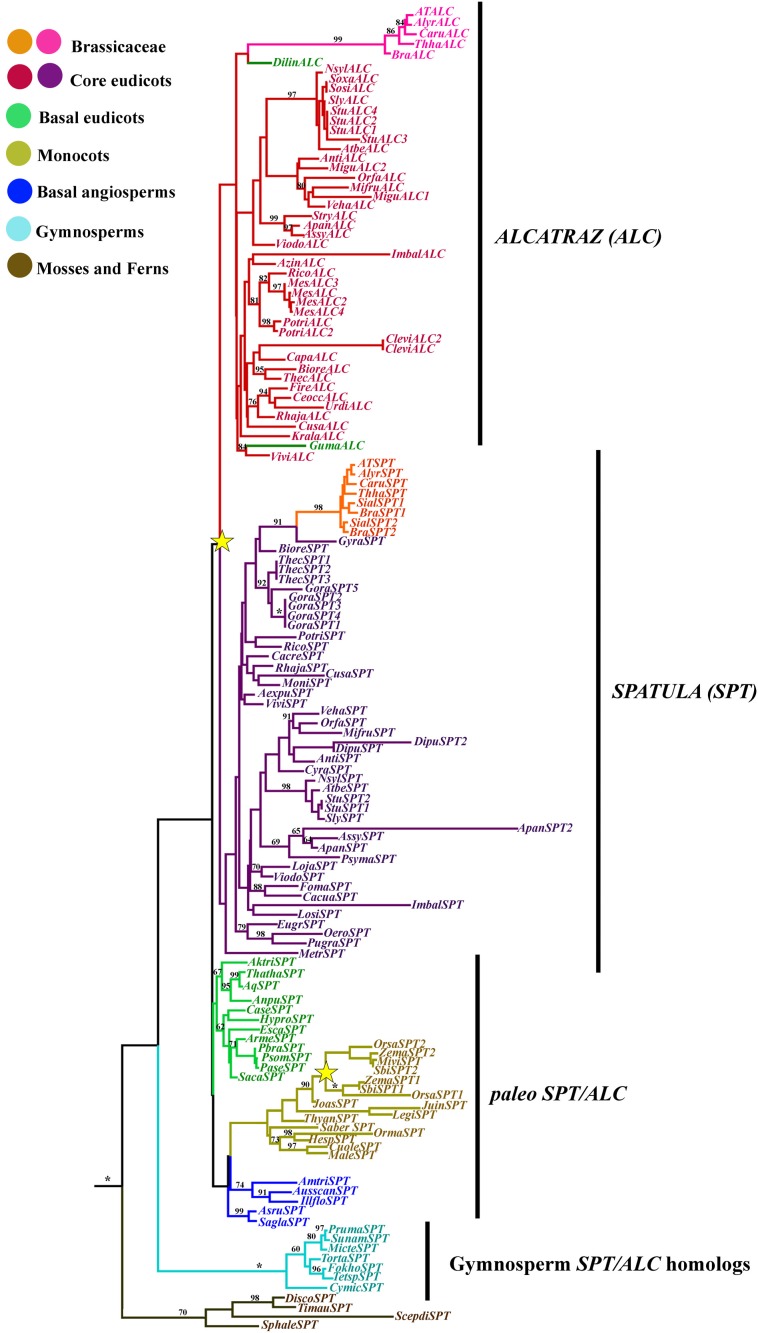
**ML tree of *SPATULA/ALCATRAZ* genes in seed plants showing two duplication events (yellow stars)**. One duplication in the Poaceae, resulting in two *SPATULA-like* clades, and a second independent duplication coincident with the diversification of the core eudicots resulting in the *SPT* and the *ALC* clades. Most sequence changes are linked with the *ALC* genes, particularly in Brassicaceae. Branch colors denote taxa as per color key at the top left; BS above 50% are placed at nodes; asterisks indicate BS of 100.

Because the beginning of the proteins was extremely variable and the homologous nucleotides in the alignment were not clear, we only used the AA from the beginning of the bHLH domain until the end of the proteins for the phylogenetic analysis. A total of 703 characters were included in the matrix, of which 224 (32%) were informative. Maximum likelihood analysis recovered two duplication events. The most important is correlated with the diversification of the core eudicots, resulting in the SPATULA and the ALCATRAZ gene clades (Figure 7). Nevertheless, support for this duplication is extremely low (<50), likely because the bHLH motif has little variation, and positional homology cannot be assigned confidently outside this region (Toledo-Ortiz et al., 2003; Pires and Dolan, 2010). This contrasts with the single copy SPT/ALC homolog present in basal eudicots, most monocots, basal angiosperms and gymnosperms. Another duplication is again correlated with the diversification of the Poaceae (Figure 7), that also has low BS (Figure 7). However, clades resulting from this duplication have BS100. Most core eudicots had at least two copies, one belonging to the SPT and the other to the ALC clades, however, taxon-specific duplications of *SPT* genes were observed in *Gossypium, Theobroma* (Malvaceae), *Digitalis* (Plantaginaceae), *Solanum tuberosum* (Solanaceae), *Apocynum* (Apocynaceae), and *Brassica* (Brasssicaceae). Our analysis also detected taxon-specific duplications of *ALC* genes in *S. tuberosum* (Solanaceae), *Manihot* (Euphorbiaceae), *Populus* (Salicaceae), and *Cleome* (Cleomaceae).

Although gene losses are harder to confirm, *SPT* homologs were not found in the genome assemblies of *Manihot* (Euphorbiaceae), *Carica* (Caricaceae), and *Mimulus* (Phrymaceae), or the transcriptomic sequences available for: *Urtica* (Urticaceae), *Celtis* (Ulmaceae), *Ficus* (Moraceae), *Cleome* (Cleomaceae), *Strychnos* (Loganiaceae), *Azadirachta* (Meliaceae). On the other hand *ALC* homologs were not found in the genomic sequences available for *Medicago* (Fabaceae), *Eucalyptus* (Myrtaceae), and *Gossypium* (Malvaceae) and the transcriptomes of *Castanea* (Fagaceae), *Digitalis* (Plantaginaceae), *Punica* (Lythraceae), *Oenothera* (Oenotheraceae), *Lobelia* (Campanulaceae), *Cavendishia* (Ericaceae), and *Fouquieria* (Fouquieriaceae).

### *INDEHISCENT*/*HECATE3* gene lineage

**Figure 8:**
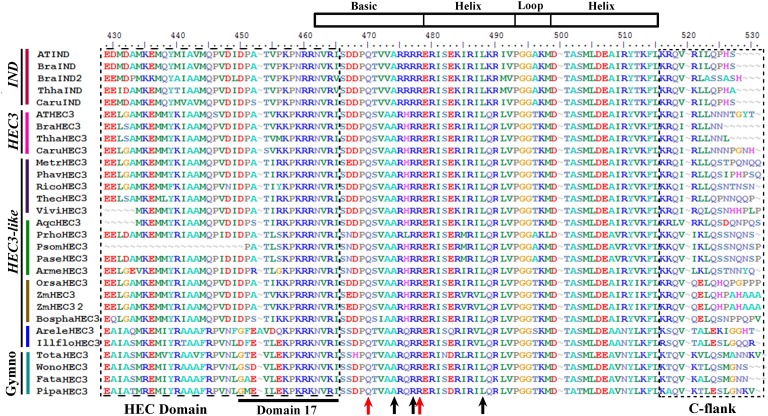
**Alignment of the bHLH domain of *HECATE3/INDEHISCENT* proteins (labeled with the clade names they belong to)**. Colors to the left of the sequences indicate the taxa they belong to as per color key in Figure 9. The bHLH was drawn based on Toledo-Ortiz et al. (2003) and in our alignment corresponds with positions N462-L515. Boxed to the left is the N-flank of the bHLH domain rich in hydrophobic aminoacids (called the HEC domain by Kay et al. (2013) and includes domain 17 by Pires and Dolan (2010); note that to Kay et al. (2013) the bHLH domain starts at S462 right after the end of the HEC domain). Black arrows in the bHLH domain indicate key aminoacids for E-box binding activity. Although R16 and L27 are conserved, position E13 (see Figure 6) is replaced by a hydrophobic A13 suggesting that HEC3/IND proteins lack this activity. Note that R17 (red arrow) is still conserved but due to the lack of E13 is unclear whether this amino acid conferring specificity plays any role in binding on its own. Additionally, the classic G-box recognition motif is not present in this proteins as the critical H/K positively changes aminoacids are replaced by Q9 with polar and uncharged side chains. Boxed to the right is the poorly conserved C flank of the bHLH motif.

INDEHISCENT (IND) and HECATE3 (HEC3) also belong to the large bHLH transcription factor family (Heim et al., 2003; Toledo-Ortiz et al., 2003). Sequences recovered by similarity in the transcriptomes generally span the entire coding sequence. The alignment of the ingroup consists of a total of 56 sequences (i.e., 5 sequences from 5 species of gymnosperms, 2 sequences from 2 species of basal angiosperms, 14 sequences from 10 species of monocots, 5 sequences from 5 species of basal eudicots, and 30 sequences from 23 species of core eudicots). The alignment includes a first region extremely variable of 415 AA, where there are very few regions of conserved amino acids and no evident conserved motifs, even in closely related taxa. This is followed by a short region rich in DE (negatively charged amino acids) until AA 430. Immediately after there is the N flank of the bHLH domain with a large region of hydrophobic amino acids from AA 430 to 449, identified previously as the HEC domain, and present only in IND/HEC3 genes when compared to other HEC genes (like HEC1 and 2) (Heim et al., 2003; Gremski et al., 2007; Pires and Dolan, 2010). This region also includes a small motif identified as conserved for all members of bHLH group VIIb called Domain 17 by Pires and Dolan (2010) (Figure 8). The end of this domain overlaps with the beginning of the basic region of the bHLH domain. Within the bHLH domain, that goes from AA 462 to 515, the IND/HEC3 proteins, as most other AtbHLH proteins, have on average 9 positively charged (K, R, and H) amino acids, in the basic motif (Figure 8) that spans 17 AA. This is followed by the completely conserved helices interrupted by a loop (HLH), responsible for homodimerization and heterodimerization (Murre et al., 1989; Ferre-D'Amare et al., 1994; Nair and Burley, 2000; Toledo-Ortiz et al., 2003; Girin et al., 2010, 2011). Unlike most other bHLH proteins studied to date, the IND/HEC3 proteins have changes in some of the key amino acids, and they possess Q9 instead of H9, A13 instead of E13, they have R16 and R17 and they also conserve L27, A39, Q56 (Figure 8). The lack of H9 and E13 suggests that IND and HEC3 are not E-box binders (CANNTG) (Fisher and Goding, 1992; Ellenberg et al., 1994; Shimizu et al., 1997; Fuji et al., 2000; Toledo-Ortiz et al., 2003). After the end of the second helix there is the C flank without any regions obviously conserved (Figure 8). From the position 530 until the end of the alignment at AA 655 there are no other regions that seem to be conserved across all *IND/HEC3* homologs. In this region, there is a very noticeable increase in the variation and shortening of the coding sequence in the Brassicaceae IND homologs suggesting a faster sequence change likely linked with divergent functions in this gene clade compared with other angiosperm and gymnosperm IND/HEC3 proteins.

**Figure 9:**
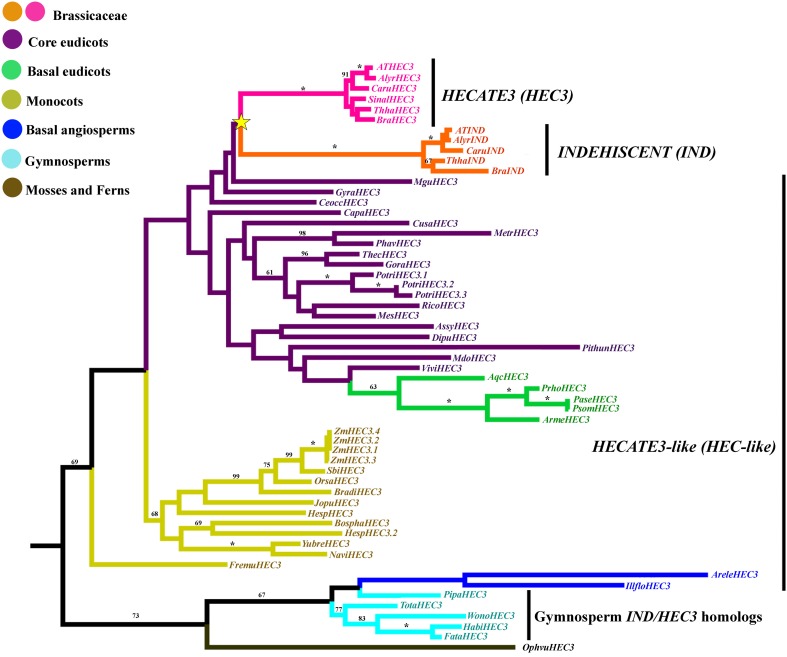
**ML tree of *INDEHISCENT/HECATE3* genes in seed plants showing a duplication in Brassicaceae (yellow star)**. This duplication resulted in the *INDEHISCENT* Brassicaceae specific genes from a HECATE3-like ancestral single copy in most core and basal eudicots, monocots and basal angiosperms. Most sequence changes are linked with the *IND* genes. Branch colors denote taxa as per color key at the top left; BS above 50% are placed at nodes; asterisks indicate BS of 100.

Similar to the SPT/ALC proteins the IND/HEC3 presented very variable 5' and 3' sequence proteins, nevertheless the IND/HEC3 are smaller and the regions with uncertainty in the alignment were short so we decided to use the entire alignment for phylogenetic analysis. A total of 2127 characters were included in the matrix, of which 997 (47%) were informative. Maximum likelihood analysis recovered a single duplication event concordant with the origin of the Brassicaceae (Figure 9). Although BS is low, the clades resulting from this duplication have 100BS. This contrasts with the single copy IND/HEC3 homologs present in the rest of the core eudicots, basal eudicots, most monocots (with the exception of *Zea mays* that has four HEC3 paralogs), basal angiosperms and gymnosperms. Because of similarity sequences with HEC3, more noticeable before the HEC domain (data not shown) they have been called HEC3-like (Kay et al., 2013). Most core eudicots that have genomic sequences available had a single HEC3 copy with the exception of *Populus* (Salicaceae) with three paralogs. From those species with available genomic sequences we could not find homologs in *Eucalyptus* (Myrtaceae), *Manihot* (Euphorbiaceae), or *Glycine* (Fabaceae).

### *REPLUMLESS*/*POUND*-*FOOLISH* gene lineage

**Figure 10:**
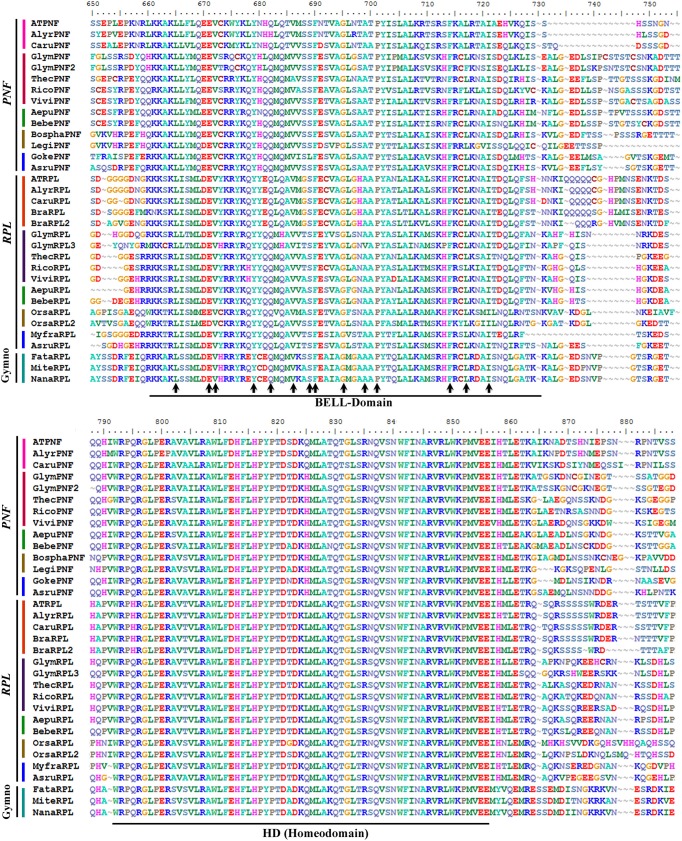
**Alignment of the BELL-domain and the Homeodomain of *REPLUMLESS*/*POUNDFOOLISH* proteins (labeled with the clade names they belong to)**. Colors to the left of the sequences indicate the taxa they belong to as per color key in Figure 11. Two domains are shown: the BELL domain (also called the MEINOX domain by Smith et al., 2002) has some invariant amino acids (arrows) in all gymnosperm and angiosperm RPL/PNF, important for dimerization that include L5, E11, V12, Y19, Q22, V26, S29, F30, G35, A40, P42, F55, L58, I62. The Homeodomain (HD) is very conserved (85%) with 53 AA conserved in seed plants out of 62 aminoacids total in the domain. Domains were drawn based on Mukherjee et al. (2009).

REPLUMLESS (RPL) and POUNDFOOLISH (PNF) belong to the TALE group of homeodomain protein (Kumar et al., 2007; Mukherjee et al., 2009) Sequences recovered by similarity in the transcriptomes generally span the entire coding sequence. The alignment of the ingroup consists of a total of 132 sequences (i.e., 11 sequences from 11 species of gymnosperms, 7 sequences from 6 species of basal angiosperms, 14 sequences from 10 species of monocots, 17 sequences from 15 species of basal eudicots, and 83 sequences from 46 species of core eudicots). The alignment includes a first region extremely variable of 544 AA with almost no similarity except sometimes in short regions between closely related taxa. Between positions 545 and 579 AA a first region of high similarity is found. This region includes a previously undescribed G/VPLF/LGPFTGYAS/TI/VLKG/SAT motif. From 560 to 575 AA a SKY motif (SKYLKPAQQ/MV/LLEEFCD/S/N) follows (Mukherjee et al., 2009), however, a true SKY motif is only present in the gymnosperm RPL/PNF proteins as in the angiosperm RPL and PNF proteins this motif is replaced by SK/RF, with the only exception being *Ascarina* (Chloranthaceae) lacking the entire motif (not shown). There is another region of high variability from AA 576 to 659 before the beginning of the 60AA BELL-domain (from AA 660 to 729) that is highly conserved across gymnosperm and angiosperm RPL/PNF proteins (Figure 10). Between the BELL-domain and the homeodomain, there is a region spanning AA 730–792 with high variability where no clear motifs can be identified. This is immediately followed by the 63AA homeodomain spanning the AA 793–856 (Figure 10). From AA 857 to 1143 there are some regions that show enough similarity to be confidently aligned, nevertheless, it is clear that there has been increased divergence in the PNF angiosperm proteins when compared to the RPL and RPL/PNF homologs in angiosperms and gymnosperms, respectively. Within this final portion of the protein the only other motif that is invariant across all RPL/PNF proteins is the “ZIBEL” motif (G/A VSLTLGL; Mukherjee et al., 2009), in our alignment located between positions 1055 and 1063 AA, at the C-terminal portion after the homeodomain. There was however no evidence in our alignment of the presence of another “ZIBEL” motif between the SKY motif and the BELL-domain, unlike what is reported in AtBEL1 and other BEL-like homeodomain proteins (Mukherjee et al., 2009).

**Figure 11:**
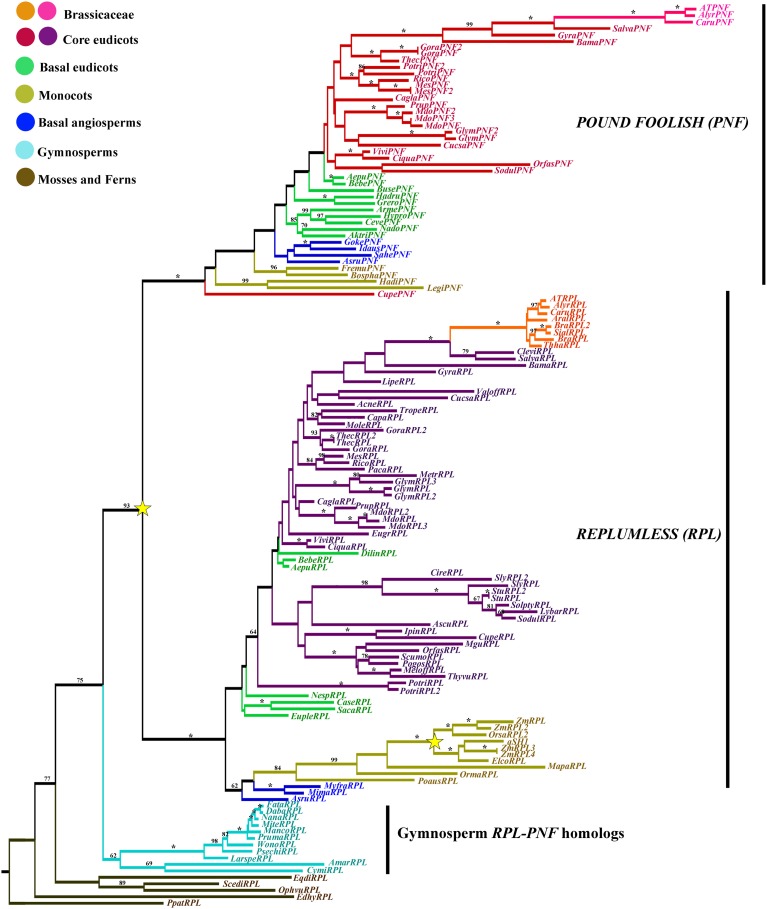
**ML tree of *REPLUMLESS/POUNDFOOLISH* genes in seed plants showing two duplications (star)**. One coinciding with the origin of the flowering plants, resulting in the *RPL* and the *PNF* clades. A second one occurring before the diversification of Poaceae. Branch colors denote taxa as per color key at the top left; BS above 50% are placed at nodes; asterisks indicate BS of 100.

A total of 2149 characters were included in the matrix, of which 757 (35%) were informative. Maximum likelihood analysis recovered a major duplication event concordant with the diversification of angiosperms resulting in the *RPL* clade and the *PNF* clade (BS 93 for the duplications and 100BS for each clade) (Figure 11). In addition a second duplication event within the *RPL* clade is evident in grasses (Poaceae). Thus, most angiosperms, except grasses, have two homologs one in each clade contrasting with the single copy *RPL/PNF* present in gymnosperms (Figure 11). Taxon-specific duplications in the *RPL* clade have occurred in *Populus* (Salicaceae), *Gossypium, Theobroma* (Malvaceae), *Solanum* (Solanaceae), *Malus* (Rosaceae), and *Glycine* (Fabaceae). On the other hand, taxon-specific duplications in the *PNF* clade include those seen in *Populus* (Salicaceae), *Glycine* (Fabaceae), *Manihot* (Euphorbiaceae), *Malus* (Rosaceae), and *Gossypium* (Malvaceae).

Although gene losses are harder to confirm, *PNF* homologs were not found in the genome assemblies of *Mimulus* (Phrymaceae), *Eucalyptus* (Myrtaceae), *Medicago* (Fabaceae), *Solanum tuberosum* and *S*. *lycopersicum* (Solanaceae), or the transcriptomic sequences available for the core eudicots: *Ipomoea* (Convolvulaceae)*, Asclepia* (Asclepiadaceae)*, Thymus, Melissa, Pogostemon, Scutellaria* (Lamiaceae)*, Moringa* (Moringaceae). *RPL* homologs were not found in the transcriptomes of several basal eudicots including: *Argemone, Hypecoum, Ceratocapnos* (Papaveraceae)*, Nandina* (Berberidaceae), and *Akebia* (Lardizabalaceae)*. One* thing to note is that no *PNF/RPL* homologs were found in *Papaver, Eschscholzia* (Papaveraceae), or *Aquilegia* (Ranunculaceae). In these taxa the similarity searches resulted in gene homologs more closely related to the outgroup sequences *SAW-like1* and *SAW-like2* than to *RPL/PNF*, although specific losses are hard to assess it is clear that at least in the *Aquilegia* genome there are no other sequences that show more similarity to *RPL/PNF* suggesting that there has been a specific loss of these genes. In the other taxa it is possible that as more transcriptomic sequences become available, *RPL/PNF* copies can be found.

## Discussion

Our data, which includes sampling from all genomes available through Phytozome and transcriptomes available in the oneKP, and the phytometasyn public blast portals allowed us to identify major duplications and losses in *AP1/FUL, STK/AG, SPT/ALC, HEC3/IND*, and *RPL/PNF* genes. Based on our analyses we have also extrapolated how the fruit developmental network as we know it from *Arabidopsis thaliana* may have evolved and been co-opted across angiosperms. Our data shows that major duplications in all gene lineages studied here coincide with paleo-polyploidization events that have been previously identified at different times in land plant evolution, namely, ε mapped to have occurred before the diversification of the angiosperms, two consecutive events known as the σ and the ρ, that occurred before the diversification of the Poaceae (Jiao et al., 2011), an independent genome-wide polyploidization event in the Ranunculales (Cui et al., 2006), the γ event at the base of the core eudicots (Jiao et al., 2011; Zheng et al., 2013), and the taxa-specific α and β duplications in lineages like the Brassicaceae, Fabaceae, and Salicaceae (Blanc et al., 2003; Bowers et al., 2003; Barker et al., 2009; Abrouk et al., 2010; Donoghue et al., 2011). Taxa-specific duplications were found frequently (in at least two of the five gene families) in *Eucalyptus* (Myrtaceae), *Glycine* (Fabaceae), *Gossypium* (Malvaceae), *Malus* (Rosaceae), *Populus* (Salicaceae), *Solanum* (Solanaceae), and *Theobroma* (Malvaceae). This is likely the result of taxon specific recent WGD as these are well-known polyploids with diploid sister groups that have retained single copy genes (Sterck et al., 2005; Sanzol, 2010; Schmutz et al., 2010; Argout et al., 2011; Grattapaglia et al., 2012; Tomato Genome Consortium, 2012). Some groups show additional gene duplications in a single gene family but not in others, for example *Manihot* (with 4 *ALC* copies), *Portulaca* and *Silene* (with 2 *euFUL* copies). These cases suggest that at least some copies may have originated by tandem repeats or retrotransposition instead of WGD or alternatively that heterogeneous diploidization events can be occurring after polyploidization (Fregene et al., 1997; Olsen and Schaal, 1999: Abrouk et al., 2010), however, assessing taxa specific duplications and losses at the family level (and infra-familial levels) will require a more comprehensive search utilizing all available EST databases as well as targeted cloning efforts.

### The MADS–box genes have undergone independent and overlapping duplication events at distinct times during plant evolution

The MADS-box genes, greatly diversified in plant evolution have been well-studied in terms of their duplications during land plant evolution (Becker and Theissen, 2003). The *AP1/FUL* lineage for instance, appeared together with the radiation of angiosperms and has duplicated independently twice in monocots (specifically Poaceae; Preston and Kellogg, 2006), once in basal eudicots (Pabón-Mora et al., 2013b) and twice in core eudicots and one additional time in Brassicaceae (Figure 3; Litt and Irish, 2003; Shan et al., 2007). All of these duplications coincide with polyploidization events previously mentioned (Blanc et al., 2003; Bowers et al., 2003; Cui et al., 2006; Barker et al., 2009; Donoghue et al., 2011; Jiao et al., 2011; Zheng et al., 2013). As a consequence of the numerous duplications, *Arabidopsis* has four gene copies: *APETALA1, CAULIFLOWER, FRUITFULL* functioning redundantly in flower meristem identity (Ferrándiz et al., 2000b), and independently in floral organ identity, specifically sepal and petal identity (*AP1, CAL*) (Coen and Meyerowitz, 1991; Bowman et al., 1993; Kempin et al., 1995; Mandel and Yanofsky, 1995) and fruit wall development (*FUL*) (Gu et al., 1998). The fourth copy, *AGAMOUS-like79* (*AGL79*) likely functioning in root development (Parenicová et al., 2003). Other core eudicots have *euAP1* genes often controlling floral meristem identity and sepal identity (Huijser et al., 1992; Berbel et al., 2001, Benlloch et al., 2006), *euFULI* genes controlling fruit wall patterning, in dry and fleshy fruits (Müller et al., 2001; Jaakola et al., 2010; Bemer et al., 2012), and *euFULII* genes (*AGL79* orthologs) playing roles in inflorescence architecture (Berbel et al., 2012). In addition some *euFULI* genes also control branching, flowering time and leaf morphology (Immink et al., 1999; Melzer et al., 2008; Berbel et al., 2012; Burko et al., 2013). Basal eudicots and monocots have a single type of gene, also referred to as the pre-duplication genes more similar to euFUL proteins, hence called *FUL-like* (Litt and Irish, 2003; Pabón-Mora et al., 2013b). Those perform a wide array of functions from leaf morphogenesis, to flowering time and transition to reproductive meristems, to sepal and sometimes petal development, to fruit wall development (Murai et al., 2003; Pabón-Mora et al., 2012, 2013a,b).

Overall, the role of *AP1/FUL* homologs in fruit development, has been recorded for many *euFUL* genes in the core eudicots and some *FUL-like* genes in basal eudicots. These analyses suggest that *euFUL* genes control proper identity and development of the fruit wall in dry fruits like that of *Antirrhinum* (Müller et al., 2001), *Nicotiana* (Smykal et al., 2007), *Arabidopsis* (Gu et al., 1998), and *Brassica* (Østergaard et al., 2006), as well as proper firmness, coloration, and ripening in fleshy fruits like that of tomato (Bemer et al., 2012; Fujisawa et al., 2014), Bilberry (Jaakola et al., 2010), peach (Tani et al., 2007; Dardick et al., 2010), and even fruits resulting from fusion of accessory organs like apple (Cevik et al., 2010). The roles in fruit development are conserved in the pre-duplication *FUL-like* genes in Papaveraceae, in the basal eudicots, where *FUL-like* genes control proper fruit wall growth, vascularization, and endocarp development (Pabón-Mora et al., 2012). Altogether the available data suggest that euFUL and FUL-like proteins act as major regulators in late fruit development that control both dehiscence and ripening and seem to have acquired these roles early on in the evolution of the angiosperms, at least before the diversification of the eudicots (see also Ferrándiz and Fourquin, 2014). Our gene tree analyses show that FUL-like proteins are present in basal angiosperms, nevertheless, because of the lack of means to down-regulate genes in basal angiosperms, there are no known roles of *FUL-like* genes in this plant group. Expression patterns are similar to those reported in basal eudicots (unpublished data), suggesting that fruit development roles are likely to be conserved in early diverging angiosperms, together with pleiotropic roles in leaf and flower development, similar to those observed in basal eudicots (Pabón-Mora et al., 2012, 2013a).

The *AG/STK* lineage is present in seed plants and duplicated at the base of flowering plants resulting in the *STK* and the *AG/SHP* clades (Figure 5; Kramer et al., 2004; Zahn et al., 2006). This duplication coincides with the ε ancestral whole genome duplication before the diversification of the angiosperms (Jiao et al., 2011). Independently, each gene clade has duplicated in monocots (Dreni and Kater, 2014). Additionally the *AG/SHP* genes (also called C-lineage or AG lineage) underwent duplications in basal eudicots (at least in Ranunculaceae), core eudicots, and the Brassicaceae, the last two coincident with the same polyploidization events γ and α/β described before (Figure 5; Blanc et al., 2003; Bowers et al., 2003; Barker et al., 2009; Donoghue et al., 2011; Jiao et al., 2011). The *STK* gene clade (also called D lineage or AGL11 lineage) has remained as single copy in all angiosperms, with the exception of grasses.

Consequently, *Arabidopsis* has four gene copies: *SEEDSTICK, AGAMOUS, SHATTERPROOF1* (*SHP1*) and *SHP2*. All four paralogs function redundantly in ovule development in *Arabidopsis* (Favaro et al., 2003; Pinyopich et al., 2003) with *SEEDSTICK* controlling also proper fertilization and seed development (Mizzotti et al., 2012)*. AGAMOUS*, represents the canonical C-function of the ABC model of flower development, and thus has specific roles in stamen and carpel identity. Finally *SHATTERPROOF* genes antagonize *FUL* and give identity to the dehiscence zone during fruit development. Functional studies in homologous genes in core eudicots and monocots have identified conserved roles in ovule development for *STK* orthologs (Colombo et al., 2008). In fact, the D-class genes involved in ovule identity were postulated based on the role of *FLORAL BINDING PROTEIN 7* (*FBP7*) in Petunia, and seem to be conserved in monocots as the *osmads13* shows defects in ovule identity (Dreni et al., 2007; Colombo et al., 2008). Additionally, *SHELL*, the *STK* homolog in oil palm (*Elaeis guineensis*) has been recently linked with oil yield, produced in the outer fibrous ring surrounding the seed, likely seed derived (Singh et al., 2013). Likewise, *STK* homologs across other non-grass monocots like *Hyacinthus* shows a restricted expression to developing ovules (Xu et al., 2004). Our gene tree analyses confirms that the *STK* or D lineage has remained predominantly unduplicated during angiosperm evolution, suggesting conserved roles in ovule identity and seed development in all angiosperms. Because these genes are also present in gymnosperms, this role is likely to be the ancestral role for the gene lineage, nevertheless more expression and functional data is needed to support this hypothesis.

On the other hand, *AG/SHP* homologs have undergone different patterns of functional evolution. Many core eudicot *euAG* and *PLE/SHP* genes have overlapping early roles in reproductive organ identity (Davies et al., 1999; Causier et al., 2005; Fourquin and Ferrandiz, 2012; Heijmans et al., 2012) and only SHP genes retain late functions in fruit development, specifically in dehiscence (Fourquin and Ferrandiz, 2012) and ripening (Vrebalov et al., 2009; Giménez et al., 2010). This is likely due to overlapping spatial and temporal expression patterns of paralogous genes (see for instance Fourquin and Ferrandiz, 2012), shared protein interactions (Leseberg et al., 2008), and lower protein sequence divergence (0.7–0.87 similarity) when compared to STK proteins (0.45–0.6) (Figure 4).

Basal eudicots and monocots have only one type of *AG* genes, known as the *paleoAG* genes, that in general only play early roles in stamen and carpel identity (Dreni et al., 2007, 2013; Yellina et al., 2010; Hands et al., 2011). Interestingly the basal eudicot paralogous genes that have been characterized in *Eschscholzia* and *Papaver*, are the result of a taxon-specific duplication in *Eschscholzia* and alternative splicing in *Papaver*. Both strategies seem to be common across basal eudicots, for instance, our sampling suggests that early diverging Papaveraceae and Lardizabalaceae have taxon-specific duplications producing two *AGAMOUS-like* copies, whereas subfamily Papaveroideae (*Papaver* and relatives including the polyploid *Argemone*) express alternative transcripts. There are also duplications that seem to have occurred before the diversification of other families, such as the Ranunculaceae (Figure 5). Functional characterization of these copies show that the two paralogs have overlapping and unique roles. For instance, in *Papaver somniferum* (Papaveraceae) one of the transcripts is largely involved in stamen and carpel identity whereas the second one becomes restricted to the carpel (Hands et al., 2011). Similar subfunctionalization scenarios have reported in Poaceae where paralogous copies in *Zea mays* and *Oryza sativa* have become functionally divergent, one largely involved in reproductive organ identity (*ZMM2* and *OsMADS3*) and the other mostly restricted to controlling carpel identity and floral meristem determinacy (*ZAG1* and *OsMADS58*) (Mena et al., 1996; Dreni et al., 2007, 2011). Nonetheless, the functional impact of taxon specific duplications will have to be discussed case by case, and will likely provide insights on the redundancy vs. sub- and neo-functionalization patterns in *AGAMOUS-like* paralogous copies. The lack of fruit defects in basal eudicot paleoAG mutants suggest that fruit development roles are unique to core eudicot copies and have become completely fixed in SHP duplicates in the Brassicaceae (Fourquin and Ferrandiz, 2012).

Expression patterns of *paleoAG* genes in basal angiosperms include stamens and carpels, and occasionally inner tepals (Kim et al., 2005) and suggest conserved roles in reproductive organ identity but do not exclude roles in late fruit development. Although comparative studies, are needed to understand the role of *AGAMOUS* homologs in early diverging flowering plants, the conserved expression of *AG/STK* homologs in gymnosperms (Jager et al., 2003; Carlsbecker et al., 2013) suggest that the ancestral role of the gene lineage includes ovule identity. Such a role was then kept as part of the functional repertoire in *STK* genes, and *AG* genes were likely recruited first for carpel identity in early diverging angiosperms and later on for fruit development in core eudicots (Kramer et al., 2004).

### Duplication of ALCATRAZ and SPATULA occurred at the base of the core eudicots

ALCATRAZ (ALC) belongs to the large bHLH transcription factor family (Pires and Dolan, 2010). In Arabidopsis, the most closely related bHLH protein to ALC is SPATULA (SPT). SPT orthologs have been identified across the seed plants (Groszmann et al., 2008). However, previous studies have been unable to identify additional ALC orthologs outside of the Brassicaceae (Groszmann et al., 2011). Therefore, the SPT and ALC duplication was thought to have occurred during a whole genome duplication event in the lineage leading to the Brassicaceae (Groszmann et al., 2011). Here we identified a duplication at the base of the core eudicots that led to the evolution of specific ALC and SPT lineages in the core eudicots. This duplication coincides with the γ duplication event (Jiao et al., 2011; Zheng et al., 2013). The presence of ALC orthologs across the core eudicots is surprising since it is necessary for differentiation of the separation layer in the dehiscence zone, which has been thought to be specific to the Brassicaceae (Eames and Wilson, 1928; Rajani and Sundaresan, 2001).

However, recent studies in Arabidopsis have shown that ALC and SPT are partially redundant in carpel and valve margin development (Groszmann et al., 2011). These proteins are thought to have undergone subfunctionalization as ALC has a more prominent role in the differentiation of the dehiscence zone and SPT has a more prominent role in carpel margin development. We identified paleo SPT/ALC orthologs in basal eudicots, basal angiosperms and monocots, that all have more than 6 basic residues in the basic region, which indicates that, these all have DNA binding activities (Figures 6, 7) (Toledo-Ortiz et al., 2003). In addition, the paleo SPT/ALC orthologs have conserved residues in the basic region that indicates that these recognize E-boxes in other proteins and specifically G-boxes (Figure 6) (Toledo-Ortiz et al., 2003). This indicates that paleo SPT/ALC may have similar downstream targets as Arabidopsis SPT and ALC.

Differences in SPT and ALC function may be due to different protein–protein interactions in the fruit developmental network. In Arabidopsis, SPT can interact with SPT, ALC, IND, and HEC, which are all bHLH proteins and are all generally involved in carpel margin development (Gremski et al., 2007; Girin et al., 2011; Groszmann et al., 2011). All of the SPT, ALC, and paleo SPT/ALC and gymnosperm SPT/ALC orthologs that we identified have a conserved Leu residue at position 27 that has been shown to be fundamental for dimer formation in mammals (Figure 6) (Toledo-Ortiz et al., 2003). In addition, there is a high level of conservation in the HLH domain of all the SPT, ALC and paleo SPT/ALC orthologs we identified and bHLH proteins are thought to form dimers with other members that have highly similar HLH domains. In species where only a single SPT/ALC ortholog was identified, it may form homodimers similar to SPT in Arabidopsis (Groszmann et al., 2011). SPT proteins have a conserved acidic domain and amphipathic helix N terminal to the bHLH domain, which is thought to be integral to its function in early gynoecium development (Groszmann et al., 2008, 2011). The amphipathic helix but not the acidic domain has been identified in ALC (Groszmann et al., 2008, 2011; Tani et al., 2011). We found the acidic domain to be conserved across angiosperms and gymnosperms except for the SPT-like2 grass genes and the Brassicaceae ALC genes. Functional analyses of ALC orthologs outside of the Brassicaceae will be necessary to understand how this gene acquired a role in dehiscence zone formation and to understand the evolution of the fruit network.

Both *SPT* and *ALC* share conserved atypical E-box elements in their cis-regulatory sequences (Groszmann et al., 2011). This sequence is required for SPT expression in the valve margin and dehiscence zone, however, similar expression studies are lacking in ALC. The expression of ALC in the valve margin is regulated by SHP1/2 and FUL in Arabidopsis (Liljegren et al., 2004). Although there are few functional analyses of SPT or ALC outside of Arabidopsis, recent studies in peach (*Prunus persica*) have indicated a role for the peach SPT ortholog (PPERSPT) in fruit development (Tani et al., 2011). PPERSPT was found to be expressed in the perianth, ovary and later in the margins of the endocarp where the carpels meet. PPERSPT is expressed in the region where the pit will later split. Further analyses of pre-duplication paleo SPT/ALC genes in angiosperms and SPT/ALC homologs in gymnosperms will be necessary to determine the ancestral function of these genes but it is likely these have roles in ovule development.

### INDEHISCENT orthologs are confined to the brassicaceae

INDEHISCENT (IND) is important for the development of the lignified layer and the separation layer in the valve margin of Arabidopsis fruits (Liljegren et al., 2004). IND belongs to the large family of bHLH transcription factors and is most closely related to HECATE3 (HEC3) in Arabidopsis (Bailey et al., 2003; Heim et al., 2003; Toledo-Ortiz et al., 2003). Our analyses across land plants show that the duplication of HEC3 and IND occurred in the lineage leading to the Brassicaceae as previous results indicated (Figure 9) (Kay et al., 2013). This duplication likely coincides with α and β genome duplications identified at the base of the Brassicaceae (Blanc et al., 2003; Bowers et al., 2003; Jiao et al., 2011). We found HEC3-like genes not only in angiosperms (Kay et al., 2013) but also in gymnosperms and ferns (Figure 9). These HEC3-like genes also share the N terminal domain, HEC, atypical bHLH and C terminal domains previously identified in angiosperms (Figure 8) (Kay et al., 2013). It is likely that the duplication resulting in HEC3 and IND in the Brassicaceae was integral for the evolution of the tissues specific to Brassicaceae fruits.

Evolution of the fruit developmental network involving IND may be due to changes in IND protein–protein interactions or to cis-regulatory changes affecting IND expression. IND interacts with both SPT and ALC to promote valve margin development (Liljegren et al., 2004; Girin et al., 2011). IND has not acquired new interactions with SPT as HEC1/2/3 can also interact with SPT (Gremski et al., 2007). However, it is not known if HEC1/2/3 can interact with ALC.

Expression of IND is found early in carpel marginal tissues and throughout the replum (Girin et al., 2011). HEC1/2/3 are also expressed in carpel marginal tissues (Gremski et al., 2007). Expression of IND later becomes restricted to the valve margin where it has a prominent role in lignification and separation layer development necessary for dehiscence (Liljegren et al., 2004; Girin et al., 2011). Sequence analyses of *Brassica rapa* IND (BraA.IND.a) and Arabidopsis IND identified a shared 400 bp sequence in the cis-regulatory regions with high similarity (Girin et al., 2010). This region was able to direct expression in the valve margin and its expression was regulated by FUL and SHP1/2 (Liljegren et al., 2000, 2004; Ferrándiz et al., 2000a; Girin et al., 2010). It is likely that this 400 bp region in the cis-regulatory region of Brassicaceae INDs was integral for the neofunctionalization of IND in dehiscence zone development.

### REPLUMLESS orthologs diversified in the angiosperms

REPLUMLESS (RPL) belongs to the TALE class of homeodomain proteins closely related to BELL (Roeder et al., 2003; Hake et al., 2004). This group of proteins has been termed BELL-Like homeodomain (BLH) proteins and have a homeodomain near the C terminus and a MEINOX INTERACTING DOMAIN (MID) near the N terminus (Hake et al., 2004; Hay and Tsiantis, 2009). The MID domain is composed of the SKY and BEL domains, which has also been largely defined as a bipartite BEL domain (Figure 10; Mukherjee et al., 2009). The MID domain, as its name indicates, is important for interacting with the MEINOX domain of the other class of TALE homeodomain proteins, KNOX. Heterodimers between KNOX and BLH are thought to give them specificity in their developmental roles. There are 13 BLH proteins in Arabidopsis and the most closely related paralog to RPL in Arabidopsis is PNF (Hake et al., 2004).

We identified PNF and RPL orthologs throughout the angiosperms indicating that a duplication occurred at the base of the angiosperms before they diversified (Figure 11). RPL is integral for replum formation in the Arabidopsis fruit and represses SHP1/2 (Roeder et al., 2003). However, RPL [also called PENNYWISE (PNY), BELLRINGER (BLR), and VAAMANA] has multiple roles in Arabidopsis development including meristem development, inflorescence, and fruit development (Byrne et al., 2003; Roeder et al., 2003; Smith and Hake, 2003; Bhatt et al., 2004; Hake et al., 2004). Therefore, it is difficult to extrapolate possible roles for the RPL orthologs that we identified. In Arabidopsis, RPL represses SHP1/2 to keep valve margin identity to a few cell layers (Roeder et al., 2003). These cell layers later become lignified and are important for fruit dehiscence. Interestingly, a RPL ortholog in rice (qSH1) is responsible for seed shattering. Grains have a lignified layer at the base where the grains will abscise at maturity. In rice, qSH1 is mutated and this is correlated with a loss of seed shattering in domesticated rice (Konishi et al., 2006; Arnaud et al., 2011). In Arabidopsis, RPL represses SHP1/2, which are the paralogous lineage of AGAMOUS (AG) (Roeder et al., 2003; Kramer et al., 2004; Zahn et al., 2006). In addition, BLR (RPL) represses AG in inflorescences and floral meristems (Bao et al., 2004). This may be an ancient regulatory module that was co-opted for carpel development in angiosperms. Analyses of RPL orthologs and their interacting KNOX proteins outside of the Brassicaceae are necessary to understand the role of RPL in fruit development and how the Arabidopsis network evolved to include RPL.

### Evolution of the fruit developmental network

**Figure 12:**
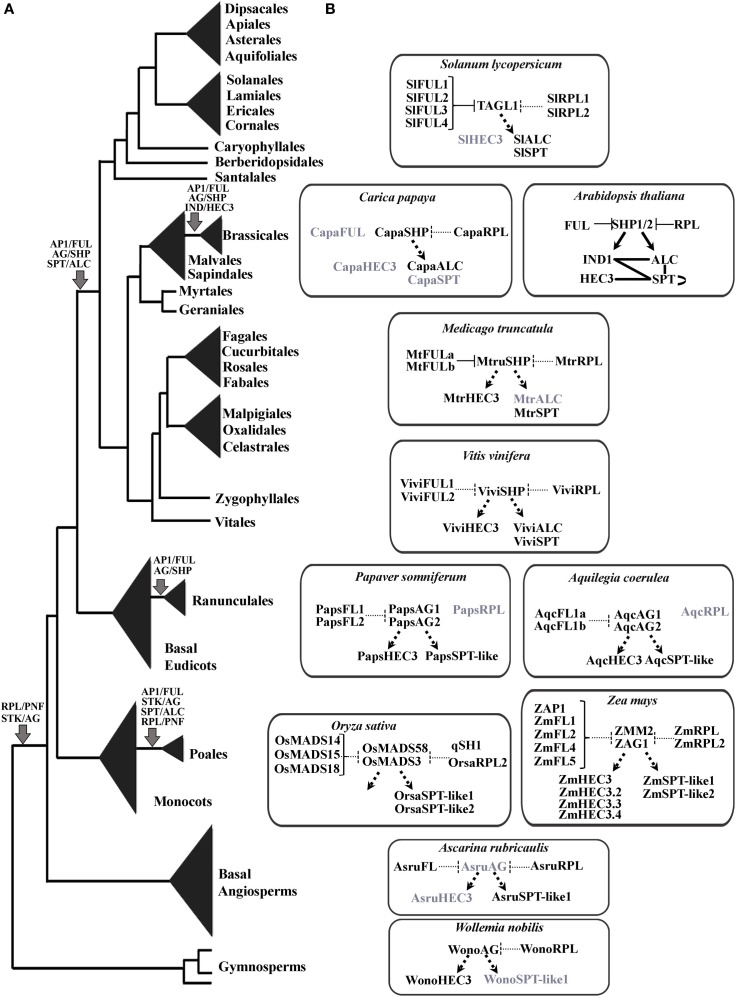
**Overview of the fruit developmental gene network**. **(A)** Seed plant phylogeny with the time points for the AP1/FUL, STK/AG, SPT/ALC, HEC3/IND, and RPL/PNF gene lineages duplications. **(B)** Reconstruction of the fruit developmental network across selected angiosperms. The only network functionally characterized is that of Brassicaceae where FUL and RPL repress SHP1/2 to shape the fruit wall, and SHP1/2 activate IND, SPT, and ALC to form the dehiscence zone. All other networks are extrapolated from *Arabidopsis*. Functional and protein–protein interaction data are necessary to validate these hypothetical interactions. Proteins in black are those previously identified or recovered in our analyses. Proteins in gray were not recovered from databases and may have been lost in the respective taxa. Solid black lines, validated protein–protein interactions; solid black arrows, validated activation; solid T-bars, validated repression; dashed lines, putative protein–protein interactions; dashed arrows, putative activation interactions; dashed T-bars, putative repression.

We have shown that the proteins involved in the Arabidopsis fruit regulatory network, namely FRUITFULL, SHATTERPROOF, REPLUMLESS, ALCATRAZ, and INDEHISCENT have undergone independent duplication events at distinct times during plant evolution. As a result the main regulators have changed in number, coding sequence and likely in protein interactions across angiosperms (Figure 12). Based on the reconstruction of all these gene lineages we were able to identify the presence of homologs of these genes across angiosperms. From our results it is clear that most core eudicots have a gene complement nearly similar to that present in the Brassicaceae, except for the lack of IND, and the presence of only one copy of SHP genes and not two as in Brassicaceae (Figure 12). Basal eudicots, monocots and basal angiosperms seem to have a narrower set of gene copies, as many duplications, coincide with the diversification of the core eudicots. Nevertheless, taxon specific duplications have occurred, and the effect of local duplicates may provide these lineages with some functional flexibility and opportunities for neofunctionalization and or subfunctionalization to occur.

We propose that a core developmental module consists of FUL-like, AG, RPL, HEC3, and SPTlike-1 and these were co-opted to play roles in basic fruit patterning and lignification. This is supported by the fact that many of the derived MADS box proteins retain early roles in carpel development, for example SHP1/2 are also involved in carpel fusion and transmitting tract development (Colombo et al., 2010). Similarly, the bHLH proteins, are important for carpel meristem development, for the development of common carpel structures such as the transmitting tract, septum and style (Groszmann et al., 2008, 2011; Girin et al., 2011). In addition, RPL is also known to have pleiotropic effects in plant development particularly in various plant meristems (Byrne et al., 2003; Roeder et al., 2003; Smith and Hake, 2003; Bhatt et al., 2004; Hake et al., 2004; Smith et al., 2004). Many of the MADS-box protein homologs present in basal angiosperms, monocots, and basal eudicots play pleiotropic functions that include floral meristem and perianth identity (e.g., AP1/FUL proteins; Bowman et al., 1993; Gu et al., 1998; Ferrándiz et al., 2000b; Berbel et al., 2001, 2012; Murai et al., 2003; Pabón-Mora et al., 2012, 2013b), ovule, stamen, and carpel identity (STK/AG proteins; Jager et al., 2003; Yellina et al., 2010; Hands et al., 2011; Carlsbecker et al., 2013).

Unraveling the evolution of the fruit developmental network may provide some insight into the evolution of the carpel, which is of great interest. Our sampling shows that basal angiosperms have the simplest network with only one gene in each gene lineage, resembling fruitless seed plants in this respect. Gymnosperms have at least one member of each gene lineage with the exception of AP1/FUL proteins. It is possible that the evolution of the AP1/FUL proteins in angiosperms was integral to the evolution of the carpel. In addition, given the pleiotropy of the core fruit module genes, comparative molecular genetic analyses of these core genes will be necessary in basal angiosperms and gymnosperms to better understand their potential roles in carpel and fruit evolution in angiosperms.

One key element to better understand the evolution of the network will be the assessment of the interactions, a poorly studied aspect, yet critical, as changes in partners between pre-duplication and post-duplication proteins may have provided core eudicots with a more robust fruit developmental network. For example, it is clear that FUL and FUL-like share a number of floral and inflorescence protein partners but it is unclear how they interact with fruit proteins (Moon et al., 1999; Ciannamea et al., 2006; Leseberg et al., 2008; Liu et al., 2010); the same has been reported for AG and SHP proteins (Leseberg et al., 2008). In addition, the bHLH proteins are known to interact with each other to regulate downstream targets (Groszmann et al., 2008, 2011; Girin et al., 2011). However, SPT is known to also form homodimers and it may be that species that we have identified with a single SPT/ALC ortholog are able to form homodimers as well but may be limited in the regulation of diverse downstream targets (Groszmann et al., 2011). The expression of ALC in the valve margin is regulated by SHP1/2 and FUL. There are shared E box elements in ALC and SPT, which are known to be important for SPT expression in valve margin (Groszmann et al., 2011). Therefore, it is likely that differences in protein interactions and their downstream targets are important for evolution of fruit network.

We have analyzed the evolution of protein families known to be the core network controlling fruit development in Arabidopsis and by doing so we have been able to identify three main lines of urgent research in fruit development: (1) The functional characterization of fruit development genes other than the MADS box members, as there are nearly no mutant phenotypes for bHLH or RPL genes outside of Arabidopsis. (2) Assessing the regulatory network by testing interactions among putative protein partners in all major groups of flowering plants to understand how the core of the ancestral fruit developmental network evolved to build fruits with diverse morphologies and (3) The morpho-anatomical detailed characterization of closely related taxa with divergent fruit types across angiosperms, to better understand what mechanisms are responsible for changes in fruit development and result in homoplasious seed dispersal syndromes, and to postulate proteins from the network likely controlling such changes.

### Conflict of interest statement

The authors declare that the research was conducted in the absence of any commercial or financial relationships that could be construed as a potential conflict of interest.
