# Supplementary material for: Evolution of fruit development genes in flowering plants
Source: Front Plant Sci. 2014 Jun 26;5:300. doi: 10.3389/fpls.2014.00300 (PMC4071287; doi:10.3389/fpls.2014.00300)
Supplement: Supplementary file 1 [file DataSheet1.DOCX]

**Supplementary Table 1.**

Accession numbers of *APETALA1/FRUITFULL* sequences used in this study.

In **bold** sequences retrieved from NCBI; *~* sequences identified through OneKp database http://www.onekp.com/; * sequences identified through Phytozome <http://www.phytozome.net/>; ^ sequences identified through AAGP (Ancestral Angiosperm Genome Project, http://ancangio.uga.edu); # sequences identified through Phytometasyn (www.phytometasyn.ca).

| Gene name | Species | Family | Accession number |
| --- | --- | --- | --- |
| ***Outgroup*** | | | |
| ***ElguiAGL6*** | *Elaeis guiineensis* | Arecaceae | AY739701 |
| ***CrosaAGL6*** | *Crocus sativus* | Iridaceae | EF041506 |
| ***MagraAGL6*** | *Magnolia grandiflora* | Magnoliaceae | AY936233 |
| ***AqAGL6*** | *Aquilegia coerulea* | Ranunculaceae | JX680248 |
| ***PehyAGL6*** | *Petunia hybrida* | Solanaceae | AB031035 |
| *DriwinAGL6*~ | *Drymis winteri* | Winteraceae | WKSU-2023594 |
| ***Ingroup*** | | | |
| **Basal Angiosperms** | | | |
| *AmtriFL1*~ | *Amborella trichopoda* | Amborellaceae | URDJ-2009015 |
| *AnmuFL1*~ | *Annona muricata* | Annonaceae | YZRI-2003332 |
| *AreleFL1*~ | *Aristolochia elegans* | Aristolochiaceae | PAWA-2045885 |
| ***AfimFL1*** | *Aristolochia fimbriata* | Aristolochiaceae | KF500113 |
| ***AcauFL1*** | *Asarum caudatum* | Aristolochiaceae | KF500105 |
| ***AeurFL1*** | *Asarum europaeum* | Aristolochiaceae | KF500104 |
| ***AeurFL2*** | *Asarum europaeum* | Aristolochiaceae | KF500103 |
| ***AeurFL3*** | *Asarum europaeum* | Aristolochiaceae | KF500107 |
| ***ShenFL1*** | *Saruma henryi* | Aristolochiaceae | KF500102 |
| *CafloFL1*~ | *Calycanthus floridus* | Calycanthaceae | FALI-2004104 |
| *AsruFL1*~ | *Ascarina rubicaulis* | Chloranthaceae | WZFE-2005264 |
| *SagraFL1*~ | *Sarcandra glabra* | Chloranthaceae | OSHQ-2046405 |
| *EubeFL1*~ | *Eupomatia bennettii* | Eupomatiaceae | DHPO-2016559 |
| *GyramFL1*~ | *Gyrocarpus americanus* | Hernandiaceae | BSVG-2010972 |
| *IllfloFL1*~ | *Illicium floridanum* | Illiciaceae | VZCI-2102640 |
| *CicaFL1*~ | *Cinnamomum camphora* | Lauraceae | BCGB-2000669 |
| ***PeamAP1*** | *Persea americana* | Lauraceae | DQ398019 |
| *PeboFL1*~ | *Persea borbonia* | Lauraceae | WIGA-2098823 |
| *PeboFL2*~ | *Persea borbonia* | Lauraceae | WIGA-2014341 |
| *SasalFL1*~ | *Sassafras albidum* | Lauraceae | ABSS-2611366 |
| ***MagrAP1*** | *Magnolia grandiflora* | Magnoliaceae | AY821777 |
| *LasemFL1*~ | *Laurelia sempervirens* | Monimiaceae | WAIL-2020232 |
| *PeuboFL1*~ | *Peumus boldus* | Monimiaceae | KRJP-2096213 |
| *MyfraFL1*~ | *Myristica fragrans* | Myristicaceae | OBPL-2044815 |
| *NuadFL1*~ | *Nuphar advena* | Nymphaceae | WTKZ-2009231 |
| *PefraFL1*~ | *Peperomia fraseri* | Piperaceae | XSZI-2000789 |
| *PefraFL2*~ | *Peperomia fraseri* | Piperaceae | XSZI-2011750 |
| ***HcAP1*** | *Houttuynia cordata* | Saururaceae | AB089153 |
| *DriwinFL1*~ | *Drymis winteri* | Winteraceae | WKSU-2033439 |
| **Monocots** | | | |
| *LegiFL1*~ | *Lepidospema gibsonii* | Cyperaceae | WBIB-2006592 |
| *BradFL1** | *Brachypodium distachyon* | Poaceae | Bradi1g59250 |
| *BradFL2** | *Brachypodium distachyon* | Poaceae | Bradi1g08340 |
| *BradFL3** | *Brachypodium distachyon* | Poaceae | Bradi1g21980 |
| *OsMADS14** | *Oryza sativa* | Poaceae | LOC-Os03g54160.1 |
| *OsMADS15** | *Oryza sativa* | Poaceae | LOC-Os07g01820.1 |
| *OsMADS18** | *Oryza sativa* | Poaceae | LOC-Os07g41370.1 |
| *ZemaFL1** | *Zea mays* | Poaceae | GRMZM2G553379.T07 |
| *ZemaFL2** | *Zea mays* | Poaceae | GRMZM2G032339.T02 |
| *ZAP1** | *Zea mays* | Poaceae | GRMZM2G148693.T02 |
| *ZemaFL4** | *Zea mays* | Poaceae | GRMZM2G072582.T02 |
| *ZemaFL5** | *Zea mays* | Poaceae | GRMZM2G147716.T01 |
| **Basal Eudicots** | | | |
| ***BebeFL1*** | *Berberis bealei* | Berberidaceae | JN593334 |
| ***BthumFL1*** | *Berberis thumbergii* | Berberidaceae | KF500149 |
| ***DpleFL1*** | *Dysosma pleiantha* | Berberidaceae | JN593333 |
| ***EsagFL1*** | *Epimedium sagittatum* | Berberidaceae | JN590216 |
| ***JediFL1*** | *Jeffersonia diphylla* | Berberidaceae | KF500146 |
| ***NdomFL1*** | *Nandina domestica* | Berberidaceae | KF500147 |
| ***NdomFL2*** | *Nandina domestica* | Berberidaceae | KF500148 |
| ***EuplFL1*** | *Euptelea pleiosperma* | Eupteleaceae | DQ656558 |
| ***EuplFL2*** | *Euptelea pleiosperma* | Eupteleaceae | DQ656559 |
| ***DexiFL1*** | *Dicentra eximia* | Fumariaceae | KF500115 |
| ***DexiFL2*** | *Dicentra eximia* | Fumariaceae | KF500155 |
| ***AktFL1*** | *Akebia trifoliata* | Lardizabalaceae | AY627632 |
| ***AktFL2*** | *Akebia trifoliata* | Lardizabalaceae | GU357459 |
| ***EscaFL1*** | *Eschscholzia californica* | Papaveraceae | HM592297 |
| ***EscaFL2*** | *Eschscholzia californica* | Papaveraceae | HM592298 |
| ***EscaFL3*** | *Eschscholzia californica* | Papaveraceae | KF500168 |
| *EscaFL4* | *Eschscholzia californica* | Papaveraceae | Submitted |
| ***LaspecFL1*** | *Lamprocapnos spectabilis* | Papaveraceae | KF500114 |
| ***LaspecFL2*** | *Lamprocapnos spectabilis* | Papaveraceae | KF500152 |
| ***MacoFL1*** | *Macleaya cordata* | Papaveraceae | KF500124 |
| ***MacoFL2*** | *Macleaya cordata* | Papaveraceae | KF500123 |
| ***MacoFL3*** | *Macleaya cordata* | Papaveraceae | KF500158 |
| ***MacoFL4*** | *Macleaya cordata* | Papaveraceae | KF500159 |
| ***MecaFL1*** | *Meconopsis cambrica* | Papaveraceae | KF500129 |
| ***MecaFL2*** | *Meconopsis cambrica* | Papaveraceae | KF500165 |
| ***PbracFL1*** | *Papaver bracteatum* | Papaveraceae | KF500131 |
| ***PbracFL2*** | *Papaver bracteatum* | Papaveraceae | KF500166 |
| ***PrhFL1*** | *Papaver rhoeas* | Papaveraceae | KF500130 |
| ***PrhFL2*** | *Papaver rhoeas* | Papaveraceae | KF500167 |
| ***PapsFL1*** | *Papaver somniferum* | Papaveraceae | AY306177 |
| ***PapsFL2*** | *Papaver somniferum* | Papaveraceae | AY306178 |
| ***RocoFL1*** | *Romneya coulteri* | Papaveraceae | KF500128 |
| ***RocoFL2*** | *Romneya coulteri* | Papaveraceae | KF500164 |
| ***AqFL1A*** | *Aquilegia coerulea* | Ranunculaceae | KF500133 |
| ***AqFL1B*** | *Aquilegia coerulea* | Ranunculaceae | KF500134 |
| ***HehyFL1*** | *Helleborus x hybrida* | Ranunculaceae | KF500135 |
| ***HehyFL2*** | *Helleborus x hybrida* | Ranunculaceae | KF500171 |
| ***HycaFL1*** | *Hydrastis canadensis* | Ranunculaceae | KF500136 |
| ***HycaFL2*** | *Hydrastis canadensis* | Ranunculaceae | KF500172 |
| ***NisaFL1*** | *Nigella sativa* | Ranunculaceae | KF500137 |
| ***NisaFL2*** | *Nigella sativa* | Ranunculaceae | KF500169 |
| ***RascFUL1*** | *Ranunculus sceleratus* | Ranunculaceae | AB473875 |
| ***RascFUL2*** | *Ranunculus sceleratus* | Ranunculaceae | AB473876 |
| ***RascFUL3*** | *Ranunculus sceleratus* | Ranunculaceae | AB473877 |
| **Core Eudicots** | | | |
| *AmcruAP1~* | *Amaranthus cruentus* | Amaranthaceae | XSSD-2002041 |
| *AmcruFUL2~* | *Amaranthus cruentus* | Amaranthaceae | XSSD-2082045 |
| *KoscoAP1~* | *Kochia scoparia* | Amaranthaceae | WGET-2011433 |
| *KoscoFUL~* | *Kochia scoparia* | Amaranthaceae | WGET-2041586 |
| *ImbaAP1~* | *Impatiens balsamina* | Balsaminaceae | JEXA-2009908 |
| *ImbaFUL~* | *Impatiens balsamina* | Balsaminaceae | JEXA-2063429 |
| *ImbaFUL2~* | *Impatiens balsamina* | Balsaminaceae | JEXA-2063430 |
| *AlyrAGL79** | *Arabidopsis lyrata* | Brassicaceae | 896948 |
| *AlyrAP1** | *Arabidopsis lyrata* | Brassicaceae | 476039 |
| *AlyrCAL** | *Arabidopsis lyrata* | Brassicaceae | 911628 |
| *AlyrFUL** | *Arabidopsis lyrata* | Brassicaceae | 950843 |
| *ATAGL79** | *Arabidopsis thaliana* | Brassicaceae | At3g30260 |
| *ATAP1** | *Arabidopsis thaliana* | Brassicaceae | At1g69120 |
| *ATCAL** | *Arabidopsis thaliana* | Brassicaceae | At1g26310 |
| *ATFUL** | *Arabidopsis thaliana* | Brassicaceae | At5g60910 |
| *BraAGL79** | *Brassica rapa* | Brassicaceae | Bra036201 |
| *BraAP1** | *Brassica rapa* | Brassicaceae | Bra004007 |
| *BraAP1.2** | *Brassica rapa* | Brassicaceae | Bra038326 |
| *BraCAL** | *Brassica rapa* | Brassicaceae | Bra011021 |
| *BraFUL** | *Brassica rapa* | Brassicaceae | Bra012997 |
| *CaruAGL79** | *Capsella rubella* | Brassicaceae | Carubv10018408m |
| *CaruAP1** | *Capsella rubella* | Brassicaceae | Carubv10022005m |
| *CaruCAL** | *Capsella rubella* | Brassicaceae | Carubv10011954m |
| *CaruFUL** | *Capsella rubella* | Brassicaceae | Carubv10027017m |
| *CapaAP1** | *Carica papaya* | Caricaceae | evm.TU supercontig-1.162 |
| *SilatAP1~* | *Silene latifolia* | Caryophyllaceae | FZQN-2001327 |
| *SilatFUL~* | *Silene latifolia* | Caryophyllaceae | FZQN-2001328 |
| *CucsaAP1** | *Cucumis sativa* | Cucurbitaceae | Cucsa113430.1 |
| *CucsaFUL** | *Cucumis sativa* | Cucurbitaceae | Cucsa327980.1 |
| *MesAP1 ** | *Manihot esculenta* | Euphorbiaceae | Cassava.4.1-029935m |
| *MesAP12** | *Manihot esculenta* | Euphorbiaceae | Cassava.4.1-031659m |
| *RicoFUL** | *Ricinus comunis* | Euphorbiaceae | 30138.t000113 |
| *GlymAP1** | *Glycine max* | Fabaceae | Glyma08g36380.1 |
| *GlymFUL** | *Glycine max* | Fabaceae | Glyma04g31847.1 |
| *GlymFUL2** | *Glycine max* | Fabaceae | Glyma06g22650.2 |
| *MetrAP1** | *Medicago truncatula* | Fabaceae | Medtr8g077590.1 |
| *MetrAP12** | *Medicago truncatula* | Fabaceae | Medtr5g049070.1 |
| *MetrFUL2** | *Medicago truncatula* | Fabaceae | Medtr7g014850.1 |
| *PhavAP1** | *Phaseolus vulgaris* | Fabaceae | Phvul008G027800 |
| *PhavFUL** | *Phaseolus vulgaris* | Fabaceae | Phvul009G203400 |
| *PhavFUL2** | *Phaseolus vulgaris* | Fabaceae | Phvul003G028100 |
| *FralaFUL1~* | *Frankenia laevis* | Frankeniaceae | WPYJ-2116716 |
| *LochiAP1~* | *Loropetalum chinense* | Hammamelidaceae | TQOO-2141311 |
| *LochiFUL~* | *Loropetalum chinense* | Hammamelidaceae | TQOO-2029423 |
| *GoraAP1** | *Gossypium raimondii* | Malvaceae | Gorai.012G185500.2 |
| *GoraFUL** | *Gossypium raimondii* | Malvaceae | Gorai.003G103000.1 |
| *GoraFUL2** | *Gossypium raimondii* | Malvaceae | Gorai.001G075800.1 |
| *ThecAP1** | *Theobroma cacao* | Malvaceae | Thecc1EG011475T3 |
| *ThecFUL** | *Theobroma cacao* | Malvaceae | Thecc1EG014345T1 |
| *ThecFUL2** | *Theobroma cacao* | Malvaceae | Thecc1EG014345T2 |
| *ThecFUL3** | *Theobroma cacao* | Malvaceae | Thecc1EG018981T1 |
| *EugrFUL** | *Eucaliptus grandis* | Myrtaceae | EugrFUL I02059 |
| *EugrFUL2** | *Eucaliptus grandis* | Myrtaceae | EgrFUL2 B00634 |
| *EugrFUL3** | *Eucaliptus grandis* | Myrtaceae | EgrAP1 K02547 |
| *OrfasAP1~* | *Orobanche fasciculata* | Orobanchaceae | VTDK-2026237 |
| *AntiAP1~* | *Antirrhinum brownblanquetti* | Plantaginaceae | YRHD-2052609 |
| *AntiFUL~* | *Antirrhinum brownblanquetti* | Plantaginaceae | YRHD-2052406 |
| ***DEFH28*** | *Antirrhinum majus* | Plantaginaceae | AY040247 |
| *DipuAP1~* | *Digitalis purpurea* | Plantaginaceae | GNRI-2023414 |
| *DipuFUL2~* | *Digitalis purpurea* | Plantaginaceae | GNRI-2022919 |
| *PooleFUL1~* | *Portulaca oleraceae* | Portulacaceae | EZGR-2014526 |
| *PooleFUL2~* | *Portulaca oleraceae* | Portulacaceae | CZJT-2011657 |
| *PosuFUL~* | *Portulaca suffruticosa* | Portulacaceae | GCYL-2042567 |
| *FraveFUL** | *Fragaria vesca* | Rosaceae | mrna26119.1v1.0 |
| *FraveFUL2** | *Fragaria vesca* | Rosaceae | mrna04228.1v1.0 |
| *MdMADS5** | *Malus domestica* | Rosaceae | MDP0000013331 |
| *MdoAP12** | *Malus domestica* | Rosaceae | MDP0000269921 |
| *MdoFUL** | *Malus domestica* | Rosaceae | MDP0000218020 |
| *MdMADS2** | *Malus domestica* | Rosaceae | MDP0000289836 |
| *PrupAP1** | *Prunus persica* | Rosaceae | Ppa010723 |
| *PpFUL** | *Prunus persica* | Rosaceae | Ppa010308m |
| *PrupFUL2** | *Prunus persica* | Rosaceae | Ppa010249m |
| *CicleAP1** | *Citrus clementina* | Rutaceae | Ciclev10032490m.g |
| *CicleAP12** | *Citrus clementina* | Rutaceae | Ciclev10032584m.g |
| *CicleFUL2** | *Citrus clementina* | Rutaceae | Ciclev10012594m.g |
| *CisiFUL2** | *Citrus sinensis* | Rutaceae | Orange1.1g030228m.g |
| *PotriFUL** | *Populus trichocharpa* | Salicaceae | Potri.012G062300.1 |
| *PotriFUL2** | *Populus trichocharpa* | Salicaceae | Potri.004G115400.1 |
| *PotriFUL3** | *Populus trichocharpa* | Salicaceae | Potri.017G099800.1 |
| *MiguAP1** | *Mimulus guttatus* | Scrophulariaceae | Mgv1a012370m |
| *MiguFUL2** | *Mimulus guttatus* | Scrophulariaceae | Mgv1a013291m |
| *SlyAP1** | *Solanum lycopersicum* | Solanaceae | Solyc05g056620.2.1 |
| *SlFUL1** | *Solanum lycopersicum* | Solanaceae | Solyc03g114830.2.1 |
| *SlFUL2** | *Solanum lycopersicum* | Solanaceae | Solyc06g069430.2.1 |
| *SlyFUL3** | *Solanum lycopersicum* | Solanaceae | Solyc02g089210.2.1 |
| *SlyFUL4** | *Solanum lycopersicum* | Solanaceae | Solyc02g065730.2.1 |
| *StuAP1** | *Solanum tuberosum* | Solanaceae | PGSC0003DMG400028358 |
| *StuAP12** | *Solanum tuberosum* | Solanaceae | PGSC0003DMT400028358 |
| *StuFUL** | *Solanum tuberosum* | Solanaceae | PGSC0003DMG400004081 |
| *StuFUL2** | *Solanum tuberosum* | Solanaceae | PGSC0003DMG400024625 |
| *StuFUL3** | *Solanum tuberosum* | Solanaceae | PGSC0003DMG400004081 |
| *StuFUL4** | *Solanum tuberosum* | Solanaceae | PGSC0003DMG400001378 |
| *ViviAP1** | *Vitis vinifera* | Vitaceae | GSVIVG0101225001 |
| *ViviFUL** | *Vitis vinifera* | Vitaceae | GSVIVG01008140001 |
| *ViviFUL2** | *Vitis vinifera* | Vitaceae | GAVIVG01036549001 |

**Supplementary Table 2.**

Accession numbers of *AGAMOUS/ SHATTERPROOF 1/2/ SEEDSTICK* sequences used in this study.

| Gene name | Species | Family | Accession number |
| --- | --- | --- | --- |
| ***Outgroup*** | | | |
| ***AthAGL12*** | *Arabidopsis thaliana* | Brassicaceae | AK228190 |
| ***MpMADS5*** | *Magnolia praecocissima* | Magnoliaceae | AB050647 |
| ***ThecAGlike*** | *Theobroma cacao* | Malvaceae | XM007020117 |
| ***AqAGL12*** | *Aquilegia coerulea* | Ranunculaceae | JX680251 |
| ***TAGL12*** | *Solanum lycopersicum* | Solanaceae | NM001246835 |
| ***VvMADS26*** | *Vitis vinifera* | Vitaceae | XM002278203 |
| ***Ingroup*** | | | |
| **Gymnosperms** | | | |
| *WonoAG~* | *Wollemia nobilis* | Araucariaceae | RSCE-2006562 |
| *MideAG~* | *Microbiota decussata* | Cupressaceae | XQSG-2053285 |
| *PapaAG~* | *Papuacedrus papuana* | Cupressaceae | OVIJ-2083454 |
| ***GinbiMADS5*** | *Ginkgo biloba* | Ginkgoaceae | GU563899 |
| *MiteAG~* | *Microcachrys tetragona* | Podocarpaceae | MHGD-2071655 |
| *SuamAG~* | *Sundacarpus amarus* | Podocarpaceae | KLGF-2089070 |
| ***PiabDAL2*** | *Picea abies* | Pinaceae | X79280.1 |
| ***PiradAG*** | *Pinus radiata* | Pinaceae | AF023615 |
| *AusspiAG~* | *Austrotaxus spicatus* | Taxaceae | BTTS-2011270 |
| *PsechiAG~* | *Pseudotaxus chienii* | Taxaceae | YLPM-2012018 |
| ***TbaccAG*** | *Taxus baccata* | Taxaceae | JF519754 |
| *TonuAG~* | *Torreya nucifera* | Taxaceae | HQQM-2014418 |
| *AthcuAG~* | *Athrotaxis cuppressoides* | Taxodiaceae | XIRK-2009770 |
| ***CryjaMADS4*** | *Cryptomeria japonica* | Taxodiaceae | HM177453 |
| **Basal Angiosperms** | | | |
| *AfimAG2^* | *Aristolochia fimbriata* | Aristolochiaceae | Aristolochiab3 c15620 |
| *AfimSTK^* | *Aristolochia fimbriata* | Aristolochiaceae | Aristolochiab3 c16533 |
| ***ShenAG*** | *Saruma henryi* | Aristolochiaceae | AY464101 |
| *AusscaSTK~* | *Austrobaileya scandens* | Austrobaileyaceae | FZJL-2164997 |
| ***ChlspiSTK*** | *Chloranthus spicatus* | Chloranthaceae | AY464099 |
| *EubeAG~* | *Eupomatia bennetti* | Eupomatiaceae | DHPO-2018932 |
| ***PeamAG1*** | *Persea americana* | Lauraceae | DQ398021 |
| ***PeamAG2*** | *Persea americana* | Lauraceae | DQ398022 |
| ***MIfiAG1*** | *Magnolia figo* | Magnoliaceae | JQ326236 |
| ***MpMADS11*** | *Magnolia praecossisima* | Magnoliaceae | AB050653 |
| ***MialAG*** | *Michelia alba* | Magnoliaceae | JQ326219 |
| *MifraAG~* | *Myristica fragrans* | Myristicaceae | OBPL-2044870 |
| *SaceAG1~* | *Saururus cernuus* | Saururaceae | OPDF-2033374 |
| **Monocots** | | | |
| *NaviAG1~* | *Narcissus viridiflorus* | Amaryllidaceae | TRRQ-2191176 |
| *NaviSTK~* | *Narcissus viridiflorus* | Amaryllidaceae | TRRQ-2019300 |
| ***ElguiAG1*** | *Elaeis guineensis* | Arecaceae | AY739698 |
| ***ElguiAG2*** | *Elaeis guineensis* | Arecaceae | AY739699 |
| *RuscusAG1~* | *Ruscus sp.* | Asparagaceae | LSJW-2096485 |
| *BroreAG~* | *Brocchinia reducta* | Bromeliaceae | BYPY-2011692 |
| *BroreSTK~* | *Brocchinia reducta* | Bromeliaceae | BYPY-2011692 |
| *MacaSTK~* | *Maianthmum canadense* | Convallariaceae | XFJG-2023121 |
| *MaiaSTK~* | *Maianthemum sp.* | Convallariaceae | RCUX-2023315 |
| *HemeAG1~* | *Hemerocallis sp.* | Hemerocallidaceae | BLAJ-2021999 |
| *HemeSTK~* | *Hemerocallis sp.* | Hemerocallidaceae | BLAJ-2004968 |
| *CurcuSTK~* | *Curculigo sp.* | Hypoxidaceae | YJUG-2140635 |
| *HespSTK~* | *Heliconia sp.* | Musaceae | XHHU-2029084 |
| *BdiAG1** | *Brachypodium distachyon* | Poaceae | Bradi2g06330.1 |
| *BdiAG2** | *Brachypodium distachyon* | Poaceae | Bradi4g40350.1 |
| *BdiAG3** | *Brachypodium distachyon* | Poaceae | Bradi2g25090.1 |
| *ElcoAG1~* | *Eleusine coracana* | Poaceae | PZAP-2009609 |
| *ElcoSTK~* | *Eleusine coracana* | Poaceae | PZAP-2061480 |
| ***OsMADS3*** | *Oryza sativa* | Poaceae | L37528 |
| ***OsMADS13*** | *Oryza sativa* | Poaceae | AF151693 |
| ***OsMADS21*** | *Oryza sativa* | Poaceae | FJ750944 |
| ***OsMADS58*** | *Oryza sativa* | Poaceae | AB232157 |
| *PamuAG1~* | *Paraneurachne muelleri* | Poaceae | XUAB-2012612 |
| *SbAG1** | *Sorghum bicolor* | Poaceae | Sb03g002525 |
| *SbAG2** | *Sorghum bicolor* | Poaceae | Sb08g006460 |
| *SbAG3** | *Sorghum bicolor* | Poaceae | Sb09g006360 |
| *ZAG2** | *Zea mays* | Poaceae | GRMZM26160687-T03 |
| *ZmSTK2** | *Zea mays* | Poaceae | GRMZM2601669-T03 |
| *ZMM2** | *Zea mays* | Poaceae | GRMZM26359952-T01 |
| *ZAG1** | *Zea mays* | Poaceae | GRMZM26052890-T01 |
| *PoausSTK~* | *Posidonia australis* | Posidoniaceae | BYQM-2002448 |
| ***LaschisAG*** | *Lacandonia schismatica* | Triuridaceae | GQ214163 |
| ***LaschisSTK*** | *Lacandonia schismatica* | Triuridaceae | GQ214164 |
| *CuoleAG1~* | *Curcuma longa* | Zingiberaceae | LEMW-2023584 |
| **Basal Eudicots** | | | |
| ***BgilAG*** | *Berberis gilgiana* | Berberidaceae | AY464106 |
| *NadoAG~* | *Nandina domestica* | Berberidaceae | YHFG-2075401 |
| ***EupleAG1*** | *Euptelea pleiosperma* | Eupteleaceae | GU357452 |
| ***EupleAG2*** | *Euptelea pleiosperma* | Eupteleaceae | GU357453 |
| ***AkquiAG*** | *Akebia quinata* | Lardizabalaceae | AY464107 |
| ***AktriAG*** | *Akebia trifoliata* | Lardizabalaceae | AY627635 |
| ***AktriSTK*** | *Akebia trifoliata* | Lardizabalaceae | AY627629 |
| ***HogrAG1*** | *Holboellia grandiflora* | Lardizabalaceae | JQ806406 |
| ***HogrAG2*** | *Holboelia grandiflora* | Lardizabalaceae | JQ806407 |
| *ArmeAG1~* | *Argemone mexicana* | Papaveraceae | BFMT-2006242 |
| *ArmeAG2~* | *Argemone mexicana* | Papaveraceae | BFMT-2012280 |
| *ArmeAG3#* | *Argemone mexicana* | Papaveraceae | Repc25491 |
| *CeveSTK~* | *Ceratocapnos vesicaria* | Papaveraceae | UDHA-2093761 |
| ***EscaAG1*** | *Eschscholzia californica* | Papaveraceae | DQ088996 |
| ***EscaAG2*** | *Eschscholzia californica* | Papaveraceae | DQ088997 |
| ***EscaAGL11*** | *Eschscholzia californica* | Papaveraceae | DQ088998 |
| *HyproSTK~* | *Hypecoum procumbens* | Papaveraceae | NMGG-2061825 |
| *PrhoAG1~* | *Papaver rhoeas* | Papaveraceae | BEKN-2018230 |
| *PrhoAG2~* | *Papaver rhoeas* | Papaveraceae | BEKN- 2018229 |
| *PrhoSTK~* | *Papaver rhoeas* | Papaveraceae | IORZ-2125324 |
| *PaseAG1~* | *Papaver setigerum* | Papaveraceae | EPRK-2005601 |
| *PaseAG2~* | *Papaver setigerum* | Papaveraceae | EPRK-2005600 |
| *PaseSTK~* | *Papaver setigerum* | Papaveraceae | STDO-2040306 |
| ***PapsAG1*** | *Papaver somniferum* | Papaveraceae | GU123602 |
| ***PapsAG2*** | *Papaver somniferum* | Papaveraceae | GU123603 |
| *PapsSTK~* | *Papaver somniferum* | Papaveraceae | RQNK-2021003 |
| ***SacaAG1*** | *Sanguinaria canadensis* | Papaveraceae | AY464097 |
| *SacaAG2~* | *Sanguinaria canadensis* | Papaveraceae | XHKT-2007933 |
| *SacaSTK~* | *Sanguinaria canadensis* | Papaveraceae | XHKT-2009840 |
| *AqAG1** | *Aquilegia coerulea* | Ranunculaceae | Aquca-136-00009.1 |
| *AqAG2** | *Aquilegia coerulea* | Ranunculaceae | Aquca-022-00039.1 |
| *HycanAG1#* | *Hydrastis canadensis* | Ranunculaceae | HCARTLPFcomp7044 |
| ***ThathAG1*** | *Thalictrum thalictroides* | Ranunculaceae | JN887118 |
| ***ThathAG2*** | *Thalictrum thalictroides* | Ranunculaceae | AY867879 |
| ***MedilSTK*** | *Meliosma dilleniifolia* | Sabiaceae | AY464105 |
| **Core Eudicots** | | | |
| *HelaAG~* | *Heracleum lanatum* | Apiaceae | CWYJ-2004811 |
| *PithunAG1~* | *Pilostyles thunbergii* | Apodanthaceae | NUJO-2006215 |
| *PithunSTK~* | *Pilostyles thunbergii* | Apodanthaceae | NJJO-2004383 |
| *AssyrSHP~* | *Asclepias syriaca* | Asclepiadaceae | YADI-2001478 |
| *AssyrSTK~* | *Asclepias syriaca* | Asclepiadaceae | YADI-2008959 |
| *ErispAG~* | *Erigeron speciosus* | Asteraceae | DESP-2019452 |
| *ErispSTK~* | *Erigeron speciosus* | Asteraceae | DESP-2103767 |
| *SilmaAG~* | *Silybum marianum* | Asteraceae | EYKJ-2002840 |
| *AlyrAG** | *Arabidopsis lyrata* | Brassicaceae | 946287 |
| *AlyrSHP1** | *Arabidopsis lyrata* | Brassicaceae | 1486333 |
| *AlyrSHP2** | *Arabidopsis lyrata* | Brassicaceae | 2321962 |
| *AlyrSTK** | *Arabidopsis lyrata* | Brassicaceae | 489841 |
| ***ATAG*** | *Arabidopsis thaliana* | Brassicaceae | AT4G18960 |
| ***ATSHP1*** | *Arabidopsis thaliana* | Brassicaceae | AT3G58780 |
| ***ATSHP2*** | *Arabidopsis thaliana* | Brassicaceae | AT2G42830 |
| ***ATSTK*** | *Arabidopsis thaliana* | Brassicaceae | AT4G09960.3 |
| *BraAG** | *Brassica rapa* | Brassicaceae | Bra012564 |
| *BraSHP1** | *Brassica rapa* | Brassicaceae | Bra003356 |
| *BraSHP2** | *Brassica rapa* | Brassicaceae | Bra2004716 |
| *BraSTK** | *Brassica rapa* | Brassicaceae | Bra 037895 |
| *CaruAG** | *Capsella rubella* | Brassicaceae | Carubv 10005558m |
| *CaruSHP1** | *Capsella rubella* | Brassicaceae | Carubv 10019520m |
| *CaruSHP2** | *Capsella rubella* | Brassicaceae | Carubv 10025002m |
| *CaruSTK** | *Capsella rubella* | Brassicaceae | Carubv10003771 |
| *ThhAG1** | *Thelungiella halophila* | Brassicaceae | Thhalv 10026567m |
| *ThhSHP1** | *Thelungiella halophila* | Brassicaceae | Thhalv10017047 |
| *ThhSHP2** | *Thelungiella halophila* | Brassicaceae | Thhalv10006196 |
| *ThhSTK** | *Thellungiela halophila* | Brassicaceae | Thhal v10028938 |
| *LosipAG~* | *Lobelia siphilitica* | Campanulaceae | IZLO-2002787 |
| *LosipSTK~* | *Lobelia siphilitica* | Campanulaceae | IZLO-2006700 |
| *PoltraAG~* | *Polansia trachysperma* | Capparaceae | QSKP-2042741 |
| *LojaAG~* | *Lonicera japonica* | Caprifoliaceae | GSZA-2009796 |
| *CapaSHP** | *Carica papaya* | Caricaceae | Evm. TU supercontig_50.73 |
| *CleviAG~* | *Cleome violaceae* | Cleomaceae | LVUS-2000317 |
| *CleviSHP~* | *Cleome violaceae* | Cleomaceae | LVUS-2001041 |
| *IppurAG~* | *Ipomoea purpurea* | Convolvulaceae | XQRV-2081601 |
| *CorneAG~* | *Coriaria nepalensis* | Coriariaceae | NNGU-2092947 |
| ***JacurAG*** | *Jatropha curcas* | Euphorbiaceae | JF343121 |
| *GlymSHP1** | *Glycine max* | Fabaceae | Glyma14g03100.1 |
| *GlymSHP2** | *Glycine max* | Fabaceae | Glyma02g45730.5 |
| *GlymSHP3** | *Glycine max* | Fabaceae | Glyma08g42300.1 |
| *GlymSTK1** | *Glycine max* | Fabaceae | Glyma 06g48270.5 |
| *GlymSTK2** | *Glycine max* | Fabaceae | Glyma 04g 43640.5 |
| *MetrAG** | *Meducago truncatula* | Fabaceae | Medtr8g087860.1 |
| ***MedSHP*** | *Medicago truncatula* | Fabaceae | JX308825 |
| *MetrSTK** | *Medicago truncatula* | Fabaceae | Medtr 3g 005530.1 |
| *GyramAG~* | *Gyrostemon ramulosus* | Gyrostemonaceae | UAXP-2012949 |
| *GyramSHP~* | *Gyrostemon ramulosus* | Gyrostemonaceae | UAXP-2002082 |
| *GyramSTK~* | *Gyrostemon ramulosus* | Gyrostemonaceae | UAXP-2014154 |
| *LigraAG~* | *Linum grandiflorum* | Linaceae | MYVH-2010389 |
| *LigraSTK~* | *Linum grandiflorum* | Linaceae | MYVH-2014650 |
| *PugraSTK~* | *Punica granatus* | Lythraceae | YNUE-2002740 |
| *GoraAG1** | *Gossypium raimondii* | Malvaceae | Gorai.N017200.1 |
| *GoraAG5** | *Gossypium raimondii* | Malvaceae | Gorai. 0126042600.1 |
| *GoraSHP** | *Gossypium raimondii* | Malvaceae | Gorai 012G042600.1 |
| *GoraSTK1** | *Gossypium raimondii* | Malvaceae | Gorai. 0096265100.1 |
| *GoraSTK2** | *Gossypium raimondii* | Malvaceae | Gorai. 0096288000.1 |
| *ThecAG** | *Theobroma cacao* | Malvaceae | Thecc 1E6029596T1 |
| *ThecSHP** | *Theobroma cacao* | Malvaceae | Thecc 1EG001841t1 |
| *ThecSTK** | *Theobroma cacao* | Malvaceae | Thecc 1EG036541t1 |
| *AzinSHP~* | *Azadirachta indica* | Meliaceae | UVDC-2006147 |
| *EucgrSTK** | *Eucaliptus grandis* | Myrtaceae | Eucgr E 02863.1 |
| *OenelAG~* | *Oenothera elata* | Oenotheraceae | WBXY-2001789 |
| *OenelSHP~* | *Oenothera elata* | Oenotheraceae | ROLB-2012307 |
| *OenelSTK~* | *Oenothera elata* | Oenotheraceae | WBXY-2088164 |
| *EpispSHP~* | *Epilobium sp.* | Onagraceae | FEDW-2008946 |
| *EpispSTK~* | *Epilobium sp.* | Onagraceae | FEDW-2008636 |
| *MafaSTK~* | *Malesherbia fasciculata* | Passifloraceae | COAQ-2050939 |
| *MguAG** | *Mimulus gutattus* | Phrymaceae | Mgu1a012605 |
| *MguSTK** | *Mimulus gutattus* | Phrymaceae | Mgv 1a013047m |
| *PhyllAG~* | *Phyllanthus sp* | Phyllanthaceae | YGAT-2002946 |
| *PhyllAG2~* | *Phyllanthus sp* | Phyllanthaceae | YGAT-2005479 |
| *PhyllSTK~* | *Phyllanthus sp.* | Phyllanthaceae | YGAT-2043702 |
| *PugraSHP~* | *Punica granatum* | Punicaceae | YNUE-2023347 |
| *MDPSHP ** | *Malus domestica* | Rosaceae | MDP0000234259 |
| *PotriAG** | *Populus trichocarpa* | Salicaceae | Potri. 011G075800.1 |
| *PotriSTK** | *Populus trichocarpa* | Salicaceae | Potri 013G104900.1 |
| *PotriSTK2** | *Populus trichocarpa* | Salicaceae | Potri 019G077200.1 |
| *LybarSHP~* | *Lycium barbarum* | Solanaceae | LWCK-2040906 |
| *LybarSTK~* | *Lycium barbarum* | Solanaceae | OSMU-2015544 |
| ***TAG1*** | *Solanum lycopersicum* | Solanaceae | L26295.1 |
| ***TAGL1*** | *Solanum lycopersicum* | Solanaceae | AY098735 |
| ***TAGL11*** | *Solanum lycopersicum* | Solanaceae | NM001247265.1 |
| *StyadAG~* | *Stylidium adnatum* | Stylidiaceae | FXGI-2052212 |
| *TropeAG~* | *Tropaeolum peregrinum* | Tropaeolaceae | MYZV-2010830 |
| *TropeSHP~* | *Tropaeolum peregrinum* | Tropaeolaceae | MYZV-2007752 |
| *ViviAG** | *Vitis vinifera* | Vitaceae | GSVIVG01000802001 |
| *ViviSHP** | *Vitis vinifera* | Vitaceae | GSVIVT01021303001 |

**Supplementary Table 3.**

Accession numbers of *SPATULA/ ALCATRAZ* *bHLH* transcription factors sequences used in this study.

| Gene name | Species | Family | Accession number |
| --- | --- | --- | --- |
| ***Outgroup*** | | | |
| ***AthPIF1*** | *Arabidopsis thaliana* | Brassicaceae | NM001202630 |
| ***AthPIF3*** | *Arabidopsis thaliana* | Brassicaceae | NM179295 |
| ***AthPIF4*** | *Arabidopsis thaliana* | Brassicaceae | NM1298622 |
| ***AthPIF5*** | *Arabidopsis thaliana* | Brassicaceae | NM115768 |
| ***AthPIL1*** | *Arabidopsis thaliana* | Brassicaceae | NM130265 |
| ***Ingroup*** | | | |
| **Bryophytes** | | | |
| *DiscoSPT~* | *Dicranium scoparium* | Dicranaceae | NGTD-2007880 |
| *ScepdiSPT~* | *Sceptridium dissectum* | Ophioglossaceae | EEAQ-2089312 |
| *SphaleSPT~* | *Sphagnum lescurii* | Sphagnaceae | GOWD-2002591 |
| *TimauSPT~* | *Timmia austriaca* | Timmiaceae | ZQRI-2005157 |
| **Gymnosperms** | | | |
| *FokhoSPT~* | *Fokienia hodginsii* | Cupressaceae | UEVI-2011728 |
| *TetspSPT~* | *Tetraclinis sp.* | Cupressaceae | CGDN-2070388 |
| *CymicSPT~* | *Cycas micholitzii* | Cycadaceae | XZUY-2049748 |
| *MicteSPT~* | *Microcachrys tetragona* | Podocarpaceae | MHGD-2012087 |
| *PrumaSPT~* | *Prumnopitys andina* | Podocarpaceae | EGLZ-2010559 |
| *SunamSPT~* | *Sundacarpus amarus* | Podocarpaceae | KLGF-2092082 |
| *TortaSPT~* | *Torreya taxifolia* | Taxaceae | EFMS-2015503 |
| **Basal Angiosperms** | | | |
| *AmtriSPT~* | *Amborella trichopoda* | Amborellaceae | URDJ-2004099 |
| *AusscanSPT~* | *Austrobaileya scandens* | Austrobaileyaceae | FZJL-2164770 |
| *AsruSPT~* | *Ascarina rubricaulis* | Chloranthaceae | WZFE-2194308 |
| *SaglaSPT~* | *Sarcandra glabra* | Chloranthaceae | QSHQ-2009866 |
| *IllfloSPT~* | *Illicium floridanum* | Illiciaceae | VZCI-2013034 |
| **Monocots** | | | |
| *SaberSPT~* | *Sabal bermudana* | Arecaceae | HWUP-2098062 |
| *LegiSPT~* | *Lepidosperma gibsonii* | Cyperaceae | WBIB-2064717 |
| *JoasSPT~* | *Joinvillea ascendens* | Joinvilleaceae | WXNT-2010203 |
| *JuinSPT~* | *Juncus inflexus* | Juncaceae | CIEA-2051856 |
| *OrmaSPT~* | *Orchidantha maxillaroides* | Lowiaceae | LSKK-2019164 |
| *MaleSPT~* | *Maranta leuconeura* | Marantaceae | JNUB-2028364 |
| *HespSPT~* | *Heliconia sp.* | Musaceae | XHHU-2030294 |
| *MiviSPT~* | *Microstegium vimieum* | Poaceae | YPIC-2016213 |
| *OrsaSPT1** | *Oryza sativa* | Poaceae | LOC-Os06g06900 |
| *OrsaSPT2** | *Oryza sativa* | Poaceae | LOC-Os02g56140 |
| *SbiSPT1** | *Sorghum bicolor* | Poaceae | Sb10g004500 |
| *SbiSPT2** | *Sorghum bicolor* | Poaceae | Sb04g036450 |
| *ZemaSPT1** | *Zea mays* | Poaceae | GRMZM2G017349 |
| *ZemaSPT2** | *Zea mays* | Poaceae | GRMZM2G030744 |
| *ThyanSPT~* | *Typha angustifolia* | Typhaceae | PPQR-2009459 |
| *CuoleSPT~* | *Curcuma longa* | Zingiberaceae | OYLU-2018981 |
| **Basal Eudicots** | | | |
| *DilinALC~* | *Dillenia indica* | Dilleniaceae | EHNF-2017021 |
| *CaseSPT~* | *Capnoides sempervirens* | Fumariaceae | AUGV-2001462 |
| *HyproSPT~* | *Hypecoum procumbens* | Fumariaceae | NMGG-2061267 |
| *GumaALC~* | *Gunnera manicata* | Gunneraceae | XMQO-2016858 |
| *AktriALC~* | *Akebia trilobata* | Lardizabalaceae | CCID-2010152 |
| *ArmeSPT~* | *Argemone mexicana* | Papaveraceae | IRAF-2111869 |
| *EscaSPT~* | *Eschscholzia californica* | Papaveraceae | EVOD-2110824 |
| *PbraSPT~* | *Papaver bracteatum* | Papaveraceae | TMWO-2128881 |
| *PaseSPT~* | *Papaver setigerum* | Papaveraceae | MLPX-2019231 |
| *PsomSPT~* | *Papaver somniferum* | Papaveraceae | RQNK-2019222 |
| *SacaSPT~* | *Sanguinaria canadensis* | Papaveraceae | XHKT-2062460 |
| *AnpuSPT~* | *Anemone pulsatila* | Ranunculaceae | UPOG-2004669 |
| *AquSPT** | *Aquilegia coerulea* | Ranunculaceae | Aquca-007-007-02 |
| *ThathaSPT~* | *Thalictrum thalictroides* | Ranunculaceae | GBVZ-2077815 |
| **Core Eudicots** | | | |
| *ApanALC~* | *Apocynum androseamifolium* | Apocynaceae | YFQX-2066339 |
| *ApanSPT~* | *Apocynum androseamifolium* | Apocynaceae | JCLQ-2010848 |
| *ApanSPT2~* | *Apocynum androseamifolium* | Apocynaceae | UFQC-2006738 |
| *AssyALC~* | *Asclepias syriaca* | Asclepiadaceae | YADI-2014020 |
| *AssySPT~* | *Asclepias syriaca* | Asclepiadaceae | YADI*-*2010022 |
| *ImbalALC~* | *Impatiens balsamifera* | Balsaminaceae | JEXA-2003941 |
| *ImbalSPT~* | *Impatiens balsamifera* | Balsaminaceae | JEXA-2060087 |
| *BioreALC~* | *Bixa orellana* | Bixaceae | KPTE-20659930 |
| *BioreSPT~* | *Bixa orellana* | Bixaceae | KPTE-2010720 |
| *AlyrALC** | *Arabidopsis lyrata* | Brassicaceae | 496889 |
| *AlyrSPT** | *Arabidopsis lyrata* | Brassicaceae | 353119 |
| ***ATALC*** | *Arabidopsis thaliana* | Brassicaceae | AT5G67110 |
| ***ATSPT*** | *Arabidopsis thaliana* | Brassicaceae | AT4G36930 |
| *BraALC** | *Brassica rapa* | Brassicaceae | Bra012133 |
| *BraSPT1** | *Brassica rapa* | Brassicaceae | Bra011740 |
| *BraSPT2** | *Brassica rapa* | Brassicaceae | Bra010591 |
| *CaruALC** | *Capsella rubella* | Brassicaceae | Carubv10028570m |
| *CaruSPT** | *Capsella rubella* | Brassicaceae | Carubv10005083m |
| *SialSPT1~* | *Sinapis alba* | Brassicaceae | VMNH-2022599 |
| *SialSPT2~* | *Sinapis alba* | Brassicaceae | VMNH-2084753 |
| *ThhaALC** | *Thellungiella halophile* | Brassicaceae | Thhalv10004889m |
| *ThhaSPT** | *Thellungiella halophila* | Brassicaceae | Thhalv10025554m |
| *LosiSPT~* | *Lobelia siphillitica* | Campanulaceae | IZLO-2061968 |
| *CleviALC~* | *Cleome violaceae* | Capparidaceae | EZXQ-2006248 |
| *CleviALC2~* | *Cleome violaceae* | Capparidaceae | LVUS-2054426 |
| *LojaSPT~* | *Lonicera japonica* | Caprifoliaceae | GSZA-2003086 |
| *ViodoALC~* | *Viburnum odoratissimum* | Caprifoliaceae | HLJG-2028505 |
| *ViodoSPT~* | *Viburnum odoratissimum* | Caprifoliaceae | HLJG-2036181 |
| *CapaALC** | *Carica papaya* | Caricaceae | Evm.supercontig21.176 |
| *CusaALC** | *Cucumis sativa* | Cucurbitaceae | Cucsa395990 |
| *CusaSPT** | *Cucumis sativa* | Cucurbitaceae | Cucsa102530 |
| *CyraSPT~* | *Cyrilla racemiflora* | Cyrillaceae | YZGX-2025255 |
| *CacuaSPT~* | *Cavendishia cuatrecasii* | Ericaceae | AVJK-2027955 |
| *MesALC** | *Manihot esculenta* | Euphorbiaceae | Cassava 4.1.- 023872 |
| *MesALC2** | *Manihot esculenta* | Euphorbiaceae | Cassava 4.1.- 024156 |
| *MesALC3** | *Manihot esculenta* | Euphorbiaceae | Cassava 4.1.- 012226 |
| *MesALC4** | *Manihot esculenta* | Euphorbiaceae | Cassava 4.1.- 024305 |
| *RicoALC** | *Ricinus comunis* | Euphorbiaceae | 30170.t000264 |
| *RicoSPT** | *Ricinus comunis* | Euphorbiaceae | 30115.t.000061 |
| *MetrSPT** | *Medicago truncatula* | Fabaceae | Medtr5g017040 |
| *CacreSPT~* | *Castanea crenata* | Fagaceae | NHUA-2002041 |
| *FomaSPT~* | *Fouquieria macdougalii* | Fouquieriaceae | YSRZ-2089912 |
| *GyraSPT~* | *Gyrostemon ramulosus* | Gyrostemonaceae | UAXP-2007849 |
| *KralaALC~* | *Krameria lanceolata* | Krameriaceae | ZHMB-2016880 |
| *MifruALC~* | *Micromeria fruticosa* | Lamiaceae | WHNV-2043030 |
| *MifruSPT~* | *Micromeria fruticosa* | Lamiaceae | WHNV-2041970 |
| *GoraSPT** | *Gossypium raimondii* | Malvaceae | Gorai.007G214800.1 |
| *GoraSPT2** | *Gossypium raimondii* | Malvaceae | Gorai.007G214800.6 |
| *GoraSPT3** | *Gossypium raimondii* | Malvaceae | Gorai.007G214800.7 |
| *GoraSPT4** | *Gossypium raimondii* | Malvaceae | Gorai.007G214800.4 |
| *GoraSPT5** | *Gossypium raimondii* | Malvaceae | Gorai.008G077300.1 |
| *ThecALC** | *Theobroma cacao* | Sterculiaceae | Thecc1EG033802t.1 |
| *ThecSPT1** | *Theobroma cacao* | Sterculiaceae | Thecc1EG000649t.1 |
| *ThecSPT2** | *Theobroma cacao* | Sterculiaceae | Thecc1EG000649t.3 |
| *ThecSPT3** | *Theobroma cacao* | Sterculiaceae | Thecc1EG000649t.2 |
| *AzinALC~* | *Azadirachta indica* | Meliaceae | UVDC-2040560 |
| *FireALC~* | *Ficus religiosa* | Moraceae | EDHN-2052162 |
| *MoniSPT~* | *Morus nigra* | Moraceae | XVJB-2054356 |
| *EugrSPT** | *Eucalyptus grandis* | Myrtaceae | Eucgr100906 |
| *OeroSPT~* | *Oenothera rosea* | Onagraceae | XSNO-2053804 |
| *OrfaALC~* | *Orobanche fasciculata* | Orobanchaceae | VYDM-2129199 |
| *OrfaSPT~* | *Orobanche fasciculata* | Orobanchaceae | VYDM-2130625 |
| *MiguALC1** | *Mimulus guttatus* | Phrymaceae | Mgv.1a010846m.g |
| *MiguALC2** | *Mimulus guttatus* | Phrymaceae | Mgv.1a015741m.g |
| *PugraSPT~* | *Punica granatum* | Punicaceae | YNUE-2022622 |
| *RhajaALC~* | *Rhamnus japonica* | Rhamnaceae | EILE-2041584 |
| *RhajaSPT~* | *Rhamnus japonica* | Rhamnaceae | EILE-2040105 |
| *PsymaSPT~* | *Psychotria marginata* | Rubiaceae | PCNH-2013819 |
| *PotriALC~* | *Populus trichocarpa* | Salicaceae | Potri014G025800 |
| *PotriALC2~* | *Populus trichocarpa* | Salicaceae | Potri002G124400 |
| *PotriSPT~* | *Populus trichocarpa* | Salicaceae | Potri005G139700 |
| *AntiALC~* | *Antirrhinum brown-blanquettii* | Scrophulariaceae | YRMD-2051629 |
| *AntiSPT~* | *Antirrhinum brown-blanquettii* | Scrophulariaceae | YRMD-2053038 |
| *DipuSPT~* | *Digitalis purpurea* | Scrophulariaceae | GNRI-2025199 |
| *DipuSPT2~* | *Digitalis purpurea* | Scrophulariaceae | GNRI-2025200 |
| *AtbeALC~* | *Atropa belladonna* | Solanaceae | BOLZ-2009637 |
| *AtbeSPT~* | *Atropa belladonna* | Solanaceae | BOLZ-2039914 |
| *NsylALC~* | *Nicotiana sylvestris* | Solanaceae | MKZR-2027110 |
| *NsylSPT~* | *Nicotiana sylvestris* | Solanaceae | MKZR-2026090 |
| *SlyALC** | *Solanum lycopersicum* | Solanaceae | Solyc03g0444460 |
| *SlySPT** | *Solanum lycopersicum* | Solanaceae | Solyc02g093280.2 |
| *SosiALC~* | *Solanum sisymbrifolium* | Solanaceae | NMDZ-2014799 |
| *StuALC1** | *Solanum tuberosum* | Solanaceae | PGSC0003DMT400020534 |
| *StuALC2** | *Solanum tuberosum* | Solanaceae | PGSC0003DMT400020533 |
| *StuALC3** | *Solanum tuberosum* | Solanaceae | PGSC0003DMT400020532 |
| *StuALC4** | *Solanum tuberosum* | Solanaceae | PGSC0003DMT400020535 |
| *StuSPT1** | *Solanum tuberosum* | Solanaceae | PGSC0003DMT4000102553 |
| *StuSPT2** | *Solanum tuberosum* | Solanaceae | PGSC0003DMT400010252 |
| *SoxaALC~* | *Solanum xanthocarpum* | Solanaceae | LQJY-2012206 |
| *StryALC~* | *Strychnos spinosa* | Strychnaceae | GGJD-2015585 |
| *AexpuSPT~* | *Aextoxilon punctatum* | Thymelaeaceae | QUTB-2009676 |
| *CeoccALC~* | *Celtis occidentalis* | Ulmaceae | KYAD-2049105 |
| *UrdiALC~* | *Urtica dioica* | Urticaceae | WKCY-2012040 |
| *VehaALC~* | *Verbena hastata* | Verbenaceae | GCFE-2051718 |
| *VehaSPT~* | *Verbena hastata* | Verbenaceae | GCFE-2054082 |
| *ViviALC~* | *Vitis vinifera* | Vitaceae | GSVIVT01009467001 |
| *ViviSPT~* | *Vitis vinifera* | Vitaceae | GSVIVG0102211001 |

**Supplementary Table 4**

Accession numbers of *INDEHISCENT1* (IND1)*/ HECATE3* (*HEC3*) sequences used in this study.

| Gene name | Species | Family | Accession number |
| --- | --- | --- | --- |
| ***Outgroup*** | | | |
| *PpatHECa** | *Physcomitrella patens* | Funariaceae | Pp 1s67-37v6 |
| *PpatHECb** | *Physcomitrella patens* | Funariaceae | Pp 1s55-11v6 |
| *PpatHECc** | *Physcomitrella patens* | Funariaceae | Pp 1s273-20v6 |
| *OphvuHEC3~* | *Ophioglossum vulvatum* | Ophioglossaceae | WTJG-2020835 |
| ***Ingroup*** | | | |
| **Gymnosperms** | | | |
| *WonoHEC3~* | *Wollemia nobilis* | Araucariaceae | RSCE-2008289 |
| *PipaHEC3~* | *Pinus parviflora* | Pinaceae | IIOL-2073963 |
| *FataHEC3~* | *Falcatifolium taxoides* | Podocarpaceae | PLYX-2077541 |
| *HabiHEC3~* | *Halocarpus bidwillii* | Podocarpaceae | OWFC-2053975 |
| *TotaHEC3~* | *Torreya taxifolia* | Taxaceae | EFWS-2079283 |
| **Basal Angiosperms** | | | |
| *AreleHEC3~* | *Aristolochia elegans* | Aristolochiaceae | PAWA-2002890 |
| *IllfloHEC3~* | *Illicium floridanum* | Illiciaceae | VZCI-2008670 |
| **Monocots** | | | |
| *YubreHEC3~* | *Yucca brevifolia* | Agavaceae | YBML-2120013 |
| *NaviHEC3~* | *Narcissus viridiflorus* | Amaryllidaceae | TRRQ-2006847 |
| *JopuHEC3~* | *Johnsonia pubescens* | Anthericaceae | WTDE-2008124 |
| *BosphaHEC3~* | *Borya sphaerocephala* | Anthericaceae | EMJJ-2021493 |
| *HespHEC3~* | *Hemerocallis sp.* | Hemerocallidaceae | BLAS-2009220 |
| *HespHEC3.2~* | *Hemerocallis sp.* | Hemerocallidaceae | JHUL-2126071 |
| *FremuHEC3~* | *Freycinetia multiflora* | Pandanaceae | DGXS-2016230 |
| *BradiHEC3** | *Brachypodium distachyon* | Poaceae | Bradi3g13300 |
| *OrsaHEC3** | *Oryza sativa* | Poaceae | LOC-Os08g01700 |
| *SbiHEC3** | *Sorghum bicolor* | Poaceae | Sb07g000900 |
| *ZmHEC3** | *Zea mays* | Poaceae | GRMZM5G818776 |
| *ZmHEC3.2** | *Zea mays* | Poaceae | GRMZM5G802883 |
| *ZmHEC3.3** | *Zea mays* | Poaceae | GRMZM2G354618 |
| *ZmHEC3.4** | *Zea mays* | Poaceae | GRMZM5G817854 |
| **Basal Eudicots** | | | |
| *ArmeHEC3~* | *Argemone mexicana* | Papaveraceae | IRAF-2030583 |
| *PrhoHEC3~* | *Papaver rhoeas* | Papaveraceae | IORZ-2009205 |
| *PaseHEC3~* | *Papaver setigerum* | Papaveraceae | QCOO-2070335 |
| *PsomHEC3~* | *Papaver somniferum* | Papaveraceae | RQNK-2056984 |
| *AqcHEC3** | *Aquilegia coerulea* | Ranunculaceae | Aquca022-00317 |
| **Core Eudicots** | | | |
| *PithunHEC3~* | *Pilostyles thunbergii* | Apodanthaceae | NJJO-2030143 |
| *AssyIHEC3~* | *Asclepias syriaca* | Asclepiadaceae | YADI-2007542 |
| *AlyrHEC3** | *Arabidopsis lyrata* | Brassicaceae | 487795 |
| *AlyrIND** | *Arabidopsis lyrata* | Brassicaceae | 490556 |
| *ATHEC3** | *Arabidopsis thaliana* | Brassicaceae | AT5G09750 |
| *ATIND** | *Arabidopsis thaliana* | Brassicaceae | AT4G00120 |
| *BraHEC3** | *Brassica rapa* | Brassicaceae | Bra028620 |
| *BraIND** | *Brassica rapa* | Brassicaceae | Bra000979 |
| *CaruHEC3** | *Capsella rubella* | Brassicaceae | Carubv10003900m |
| *CaruIND** | *Capsella rubella* | Brassicaceae | Carubv10033163m |
| *SinalHEC3~* | *Sinapis alba* | Brassicaceae | VMNH-2014550 |
| *ThhaHEC3** | *Thellungiella halophila* | Brassicaceae | Thhalv10015330m |
| *ThhaIND** | *Thellungiella halophila* | Brassicaceae | Thhalv10029526m |
| *CapaHEC3** | *Carica papaya* | Caricaceae | Evm.supercontig 46440 |
| *CusaHEC3** | *Cucumis sativa* | Cucurbitaceae | Cucsa101020 |
| *MesHEC3** | *Manihot esculenta* | Euphorbiaceae | Cassava 4.1.- 031791 |
| *RicoHEC3** | *Ricinus comunis* | Euphorbiaceae | 30136.t.000039 |
| *MetrHEC3** | *Medicago truncatula* | Fabaceae | Medtr8g093500 |
| *PhavHEC3** | *Phaseolus vulgaris* | Fabaceae | Phvul.0026271000 |
| *GyraHEC3~* | *Gyrostemon ramulosus* | Gyrostemonaceae | UAXP-2017568 |
| *GoraHEC3** | *Gossypium raimondii* | Malvaceae | Gorai.011G116100 |
| *ThecHEC3** | *Theobroma cacao* | Sterculiaceae | Thecc1EG001435t.1 |
| *MguHEC3** | *Mimulus guttatus* | Phrymaceae | Mgv.1a023913m.g. |
| *MdoHEC3** | *Malus domestica* | Rosaceae | MDP000071774 |
| *PotHEC3** | *Populus trichocarpa* | Salicaceae | Potri005G060900 |
| *PotHEC3.2** | *Populus trichocarpa* | Salicaceae | Potri007G10800 |
| *PotHEC3.3** | *Populus trichocarpa* | Salicaceae | PotriT107900 |
| *DipuHEC3~* | *Digitalis purpurea* | Scrophulariaceae | GNRI-2130670 |
| *CeoccHEC3~* | *Celtis occidentalis* | Ulmaceae | KYAD-2049669 |
| *ViviHEC3** | *Vitis vinifera* | Vitaceae | GSVIVT01026516001 |

**Supplementary Table 5**

Accession numbers of *REPLUMLESS* (*RPL*)/*POUND-FOOLISH* (*PNF*) sequences used in this study.

| Gene name | Species | Family | Accession number |
| --- | --- | --- | --- |
| ***Outgroup*** | | | |
| *EqdiRPL~* | *Equisetum diffusum* | Equisetaceae | CAPN-2007414 |
| *EdhyRPL~* | *Equisetum hymale* | Equisetaceae | JVSZ-2015211 |
| *PpatRPL~* | *Physcomitrella patens* | Funariaceae | Pp1s220-167v6 |
| *OphvuRPL~* | *Ophioglossum vulgatum* | Ophioglossaceae | WTJG-2002390 |
| *ScediRPL~* | *Sceptridium dissectum* | Ophioglossaceae | EEAQ-2011145 |
| ***Ingroup*** | | | |
| **Gymnosperms** | | | |
| *WonoRPL~* | *Wollemia nobilis* | Araucariaceae | RSCE-2009184 |
| *AmarRPL~* | *Amentotaxus argotaenia* | Cephalotaxaceae | IAJW-2003519 |
| *CymiRPL~* | *Cycas micholitzii* | Cycadaceae | XZUY-2005481 |
| *LarspeRPL~* | *Larix speciosa* | Pinaceae | WVWN-2056102 |
| *DabaRPL~* | *Dacrydium balansae* | Podocarpaceae | IZGN-2013347 |
| *FataRPL~* | *Falcatifolium taxoides* | Podocarpaceae | ROWR-2007696 |
| *MancoRPL~* | *Manoao colensoi* | Podocarpaceae | CDFR-2065625 |
| *MiteRPL~* | *Microcachrys tetragona* | Podocarpaceae | MHGD-2076247 |
| *NanaRPL~* | *Nageia nagi* | Podocarpaceae | UUJS-2009138 |
| *PrumaRPL~* | *Prumnopitys andina* | Podocarpaceae | EGLZ-2011029 |
| *PsechiRPL~* | *Pseudotaxus chienii* | Taxaceae | YLPM-2005818 |
| **Basal Angiosperms** | | | |
| *SahePNF~* | *Saruma henryi* | Aristolochiaceae | QDVW-2015102 |
| *IdausPNF~* | *Idiospermum australiense* | Calycanthaceae | WPHN-2011608 |
| *AsruPNF~* | *Ascarina rubricaulis* | Chloranthaceae | WZFE-2196459 |
| *AsruRPL~* | *Ascarina rubricaulis* | Chloranthaceae | WZFE-2018769 |
| *GokePNF~* | *Gomortega keule* | Gomortegaceae | MAQO-2119564 |
| *MimaRPL~* | *Michelia maudiae* | Magnoliaceae | XQWC-2021315 |
| *MyfraRPL~* | *Myristica fragrans* | Myristicaceae | OBPL-2006262 |
| **Monocots** | | | |
| *BosphaPNF~* | *Borya sphaerocephala* | Anthericaceae | EMJJ-2024075 |
| *LegiPNF~* | *Lepidosperma gibsonii* | Cyperaceae | WBIB-200067 |
| *MapaRPL~* | *Mapania palustris* | Cyperaceae | XPAF-2057465 |
| *OrmaRPL~* | *Orchidantha maxillaroides* | Lowiaceae | LSKK-2011206 |
| *HadiPNF~* | *Haemaria discolor* | Orchidaceae | LELS-2012821 |
| *FremuPNF~* | *Freycinetia multiflora* | Pandanaceae | DGXS-2018607 |
| *ElcoRPL~* | *Eleusine coracana* | Poaceae | TIJL-2004683 |
| *qSH1** | *Oryza sativa* | Poaceae | LOC-Os01g62920 |
| *OrsaRPL2** | *Oryza sativa* | Poaceae | LOC-Os05g38120 |
| *ZmRPL** | *Zea mays* | Poaceae | GRMZM2G074645-T01 |
| *ZmRPL2** | *Zea mays* | Poaceae | GRMZM2G125976-T01 |
| *ZmRPL3** | *Zea mays* | Poaceae | GRMZM2G154641-T01 |
| *ZmRPL4** | *Zea mays* | Poaceae | GRMZM2G154641-T02 |
| *PoausRPL~* | *Posidonia australis* | Posidoniaceae | BYQM-2012756 |
| **Basal Eudicots** | | | |
| *AepuPNF~* | *Aetoxicon punctatum* | Aetoxicaceae | QUTB-2017244 |
| *AepuRPL~* | *Aetoxicon punctatum* | Aetoxicaceae | QUTB-2010276 |
| *NadoPNF~* | *Nandina domestica* | Berberidaceae | YHFG-2013960 |
| *BusePNF~* | *Buxus sempervirens* | Buxaceae | IWHW-2017055 |
| *DilinRPL~* | *Dilenia indica* | Dileniaceae | EHNF-2021421 |
| *EupleRPL~* | *Euptelea pleiosperma* | Eupteleaceae | QTJY-2006403 |
| *BebePNF~* | *Berberidopsis beckleri* | Flacourtiaceae | HAEU-2055923 |
| *BebeRPL~* | *Berberidopsis beckleri* | Flacourtiaceae | HAEU-2011390 |
| *AktriPNF~* | *Akebia trifoliata* | Lardizabalaceae | CCID-2003816 |
| *NespRPL~* | *Nelumbo sp.* | Nelumbonaceae | FAKD-2011666 |
| *ArmePNF~* | *Argemone mexicana* | Papaveraceae | COQJ-2013028 |
| *CaseRPL~* | *Capnoides sempervirens* | Papaveraceae | AUGV-2001864 |
| *CevePNF~* | *Ceratocapnos vesicaria* | Papaveraceae | UDHA-2003225 |
| *HyproPNF~* | *Hypecoum procumbens* | Papaveraceae | NMGG-2063192 |
| *SacaRPL~* | *Sanguinaria canadensis* | Papaveraceae | XHKT-2009137 |
| *GreroPNF~* | *Grevillea robusta* | Proteaceae | GRRW-2004243 |
| *HadruPNF~* | *Hakea drupaceae* | Proteaceae | SIIK-2019060 |
| **Core Eudicots** | | | |
| *AcneRPL~* | *Acer negundo* | Aceraceae | VFFP-2012237 |
| *AscuRPL~* | *Asclepias curassavica* | Apocynaceae | DSUV-2010692 |
| *BamaPNF~* | *Batis maritima* | Bataceae | DZTK-2002437 |
| *BamaRPL~* | *Batis maritima* | Bataceae | DZTK-2007039 |
| *AlyrPNF** | *Arabidopsis lyrata* | Brassicaceae | 481709 |
| *AlyrRPL** | *Arabidopsis lyrata* | Brassicaceae | 486980 |
| ***ATPNF*** | *Arabidopsis thaliana* | Brassicaceae | At2g27990 |
| ***ATRPL*** | *Arabidopsis thaliana* | Brassicaceae | At5g0230 |
| *AralRPL~* | *Arabis alpina* | Brassicaceae | TZWR-2044377 |
| *BraRPL** | *Brassica rapa* | Brassicaceae | Bra009618 |
| *BraRPL2** | *Brassica rapa* | Brassicaceae | Bra028883 |
| *CaruPNF** | *Capsella rubella* | Brassicaceae | Carubv10024860m |
| *CaruRPL** | *Capsella rubella* | Brassicaceae | Carubv10000448m |
| *SialRPL~* | *Sinapis alba* | Brassicaceae | WMNH-2000089 |
| *ThhaRPL** | *Thellungiella halophila* | Brassicaceae | Thhalv10012876 |
| *CleviRPL~* | *Cleome violaceae* | Capparidaceae | LVUS-2010603 |
| *CapaRPL** | *Carica papaya* | Caricaceae | Evm.TU.supercontig 56.56 |
| *CaglaPNF~* | *Casuarina glauca* | Casuarinaceae | LNER-2004636 |
| *CaglaRPL~* | *Casuarina glauca* | Casuarinaceae | LNER-2006332 |
| *CupePNF~* | *Cuscuta pentagonia* | Convolvulaceae | AHRN-2009781 |
| *CupeRPL~* | *Cuscuta pentagonia* | Convolvulaceae | AHRN-2012810 |
| *IpinRPL~* | *Ipomoea indica* | Colvolvulaceae | OQBM-2015458 |
| *CucsaPNF** | *Cucumis sativus* | Cucurbitaceae | Cucsa.131240.1 |
| *CucsaRPL* | *Cucumis sativus* | Cucurbitaceae | Cucsa.175830.1 |
| *MesPNF** | *Manihot esculenta* | Euphorbiaceae | Cassava.4.1-001638m.g |
| *MesPNF2** | *Manihot esculenta* | Euphorbiaceae | Cassava.4.1-003413m.g |
| *MesRPL** | *Manihot esculenta* | Euphorbiaceae | Cassava4.1-003548m.g. |
| *RicoPNF** | *Ricinus comunis* | Euphorbiaceae | 29638.t000014 |
| *RicoRPL** | *Ricinus comunis* | Euphorbiaceae | 29973.m000390 |
| *GlymPNF** | *Glycine max* | Fabaceae | Glyma13g39901.1 |
| *GlymPNF2** | *Glycine max* | Fabaceae | Glyma12g29991.1 |
| *GlymRPL** | *Glycine max* | Fabaceae | Glyma03g17397.1 |
| *GlymRPL2** | *Glycine max* | Fabaceae | Glyma01g25710.2 |
| *GlymRPL3** | *Glycine max* | Fabaceae | Glyma18g41280.1 |
| *MetrRPL** | *Medicago truncatula* | Fabaceae | Medtr7g065050.1 |
| *GyraPNF~* | *Gyrostemon ramulosus* | Gyrostemonaceae | UAXP-2080565 |
| *GyraRPL~* | *Gyrostemon ramulosus* | Gyrostemonaceae | UAXP-2022630 |
| *MeloffRPL~* | *Melissa officinalis* | Lamiaceae | TAGM-2049017 |
| *PogosRPL~* | *Pogostemon sp* | Lamiaceae | GETL-2015293 |
| *ScumoRPL~* | *Scutellaria montana* | Lamiaceae | ATYL-2001588 |
| *ThyvuRPL~* | *Thymus vulgaris* | Lamiaceae | IYDF-2009612 |
| *LipeRPL~* | *Linum perenne* | Linaceae | XWMS-2004890 |
| *GoraPNF** | *Gossypium raimodii* | Malvaceae | Gorai009G109300.1 |
| *GoraPNF2** | *Gossypium raimodii* | Malvaceae | Gorai009G109300.3 |
| *GoraRPL** | *Gossypium raimodii* | Malvaceae | Gorai.012G108300.1 |
| *GoraRPL2** | *Gossypium raimodii* | Malvaceae | Gorai.012G108300.2 |
| *ThecPNF** | *Theobroma cacao* | Sterculiaceae | Thecc1EG042098t1 |
| *ThecRPL** | *Theobroma cacao* | Sterculiaceae | Thecc1EG021834 |
| *ThecRPL2** | *Theobroma cacao* | Sterculiaceae | Thecc1EG021834t2 |
| *MoleRPL~* | *Moringa oleifera* | Moringaceae | CZPV-2048449 |
| *EugrRPL** | *Eucaliptus grandis* | Myrtaceae | Eucgr.K03046 |
| *OrfasPNF~* | *Orobanche fasciculata* | Orobanchaceae | VYDM-2131665 |
| *OrfasRPL~* | *Orobanche fasciculata* | Orobanchaceae | VYDM-2014609 |
| *PacaRPL~* | *Passiflora caerulea* | Passifloraceae | SIZE-2017166 |
| *MdoPNF** | *Malus domestica* | Rosaceae | MDP0000596661 |
| *MdoPNF2** | *Malus domestica* | Rosaceae | MDP0000272542 |
| *MdoPNF3** | *Malus domestica* | Rosaceae | MDP0000255392 |
| *MdoRPL** | *Malus domestica* | Rosaceae | MDP0000126587 |
| *MdoRPL2** | *Malus domestica* | Rosaceae | MDP0000429775 |
| *MdoRPL3** | *Malus domestica* | Rosaceae | MDP0000209399 |
| *PrupPNF** | *Prunus persica* | Rosaceae | Ppa.001495m.g. |
| *PrupRPL** | *Prunus persica* | Rosaceae | Ppa.002604m.g. |
| *PotriPNF** | *Populus trichocarpa* | Salicaceae | Potri.009G009900 |
| *PotriPNF2** | *Populus trichocarpa* | Salicaceae | Potri.004G213300 |
| *PotriRPL** | *Populus trichocarpa* | Salicaceae | Potri.008G061000.1 |
| *PotriRPL2** | *Populus trichocarpa* | Salicaceae | Potri.010G197300 |
| *SalvaPNF~* | *Salvadora sp.* | Salvadoraceae | RTTY-2018926 |
| *SalvaRPL~* | *Salvadora sp.* | Salvadoraceae | RTTY-2018926 |
| *MguRPL** | *Mimulus guttatus* | Scrophulariaceae | Mgu.1a002791m.g |
| *LybarRPL~* | *Lycium barbarum* | Solanaceae | LWCK-2075139 |
| *SodulPNF~* | *Solanum dulcamara* | Solanaceae | GHLP-2060348 |
| *SodulRPL~* | *Solanum dulcamara* | Solanaceae | GHLP-2006410 |
| *SlyRPL** | *Solanum lycopersicum* | Solanaceae | Solyc10g086640.1 |
| *SlyRPL2** | *Solanum lycopersicum* | Solanaceae | Solyc09g011380.2 |
| *SolptyRPL~* | *Solanum ptychanthum* | Solanaceae | DLJZ-2053145 |
| *StuRPL** | *Solanum tuberosum* | Solanaceae | PGSC0003DMG400019142 |
| *StuRPL2** | *Solanum tuberosum* | Solanaceae | PGSC003DMG400049235 |
| *TropeRPL~* | *Tropaeolum peregrinum* | Tropaeolaceae | MYZV-2059470 |
| *ValoffRPL~* | *Valeriana officinalis* | Valerianaceae | FFFY-2005096 |
| *CiquaPNF~* | *Cissus quadrangularis* | Vitaceae | BGZG-2015650 |
| *CiquaRPL~* | *Cissus quadrangularis* | Vitaceae | BGZG-2013848 |
| *ViviPNF** | *Vitis vinifera* | Vitaceae | GSVIVG0102522001 |
| *ViviRPL** | *Vitis vinifera* | Vitaceae | GSVIVG01034073001 |
